# Bidentate Acyclic Diamino Carbene-Stabilized Gold Nanoparticles from Symmetric and Asymmetric Gold(I) Complexes: Synthesis, Characterization, and Catalytic Activity

**DOI:** 10.1021/acs.inorgchem.5c03050

**Published:** 2025-09-12

**Authors:** Sophie R. Thomas, Tristan T. Y. Tan, Guilherme M. D. M. Rubio, Monnaya Chalermnon, Jia Min Chin, Michael R. Reithofer

**Affiliations:** † 27258 Institute of Inorganic Chemistry, Faculty of Chemistry, University of Vienna, Währinger Str. 42, Vienna 1090, Austria; ‡ 208718 Institute of Material Research and Engineering, A*STAR (Agency for Science, Technology and Research), Singapore 138634, Singapore; § Department of Functional Materials and Catalysis, Faculty of Chemistry, University of Vienna, Währinger Str. 42, Vienna 1090, Austria

## Abstract

A series of gold nanoparticles (AuNPs) stabilized by bidentate acyclic diamino carbenes (ADCs) were synthesized via the reduction of dimeric gold­(I) complexes. The resulting ADC-AuNPs were characterized by NMR spectroscopy, UV–vis spectroscopy, X-ray photoelectron spectroscopy (XPS), transmission electron microscopy (TEM), and thermogravimetric analysis (TGA), revealing nanoparticles with a narrow size distribution and sizes ranging from 1.7 to 3.3 nm. This synthetic approach was extended to an asymmetric dinuclear ADC-gold­(I) complex, affording slightly larger AuNPs (∼4 nm) upon reduction. The ADC-AuNPs with shorter linkers exhibited significant catalytic activity for the reduction of 4-nitrophenol, demonstrating a versatile and efficient route to catalytically active gold nanoparticles stabilized by both symmetric and asymmetric ADC ligands.

## Graphical abstract

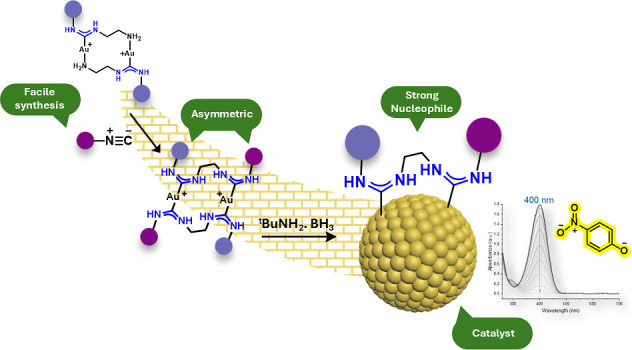

## Introduction

Over the past decades, gold nanomaterials have been extensively studied for their diverse applications in sensing,^1–4^ catalysis,^5–9^ photonics,^10–12^ imaging,^13–15^ and drug delivery.^16,17^ Significant advancements have been made in synthetic strategies to control the size and shape of gold nanomaterials.^18–21^ However, the interfacial chemistry, crucial for stabilizing these materials, has remained greatly unchanged for decades.

Strongly binding *N*-heterocyclic carbenes (NHCs, Figure 1, left), which have become ubiquitous ligands in the broader fields of chemistry, have emerged as promising alternatives to the more commonly employed but weaker-binding thiols for stabilizing gold nanomaterials.^22–26^ Given the success of NHCs, there is considerable interest in expanding the range of stable carbenes used as ligands. Acyclic diamino carbenes (ADCs) are a distinct class of stable carbenes, offering unique advantages in terms of synthetic accessibility and chemical functionality. Protic ADCs, for example, are highly accessible synthetically and can be formed easily through reactions of primary or secondary amines with isocyanide complexes (Figure 1, right). This ease of synthesis allows for the creation of metal complexes bearing asymmetric or chiral ADCs^27^ more readily than with NHCs.^28^ In addition, the straightforward formation of ADC complexes facilitates the creation of large ligand libraries, enabling tailored approaches to specific applications.^29^ Furthermore, the protons on the nitrogen atoms of protic ADCs also offer extra functionality to the ligands, such as the ability to act as hydrogen bond donors.^30^ Finally, the donor strength of ADCs is known to be stronger than NHCs, thus imparting different electronic properties to the resulting gold nanomaterials.^31^

**Figure 1:**
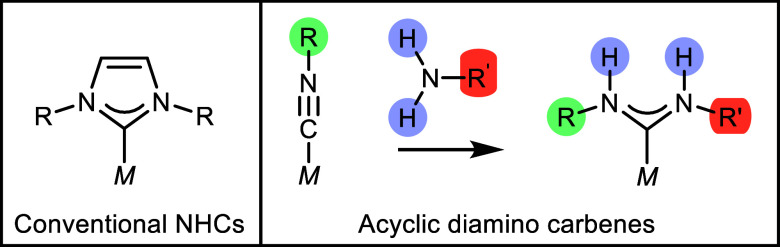
Depiction of a conventional NHC metal complex (left) and the synthesis of complexes bearing ADCs (right), achieved through the reaction of amines with coordinated isocyanides. This method facilitates straightforward access to varied R groups (highlighted in green and red) and to protons capable of hydrogen bonding (highlighted in blue).

Gold nanoparticles (AuNPs) with NHC capping ligands have been reported to catalyze various chemical reactions, including the reduction of CO_2_.^32–34^ For the catalytic study of NHC-AuNPs, the reduction of 4-nitrophenol (4-NP) to 4-aminophenol (4-AP) is also a common reaction first reported by Crespo et al.^35^ Later, Song et al., investigated the catalytic activity of (NHC)-functionalized conducting polymers with gold nanoparticles.^36^ In addition, He et al.^37^ and Han et al.^38^ also explored the catalytic activity of gold nanoparticles dispersed in a polymeric matrix. Furthermore, our group has demonstrated the use of hyper-cross-linked polymer-supported NHC-AuNPs in flow catalysis, achieving exceptional recyclability of the supported nanocatalysts.^39^ Besides this, Casini and co-workers reported a detailed investigation of water-dispersible gold nanoparticles stabilized by mono- and bidentate NHC ligands for a series of applications, including the reduction of different nitrophenol substrates.^40^

Given the success of NHC-stabilized AuNPs and the need for even greater stability, we turned our attention to bidentate ligands, which are expected to endow greater stability to nanoparticles compared to their monodentate counterparts.^24^

Herein, we report the preparation of bidentate ADC-stabilized AuNPs, expanding on our previous work where we reported the detailed syntheses of dinuclear ADC gold complexes (**a**–**e**, Scheme 1) by reacting an isocyanide gold complex with different primary diamines; the dimeric complexes were formed by coordinating one amine group of the diamine with the isocyanide to form an ADC, and the other with the gold center.^41^ In this work, the protic ADC dimeric gold complexes (**a**–**e**, Scheme 1) were reacted further with *tert*-butyl isocyanide to yield novel dinuclear gold tetrakiscarbene complexes (**1a**–**1e**, Scheme 1) before reduction to ADC-AuNPs (**1a**–**e**-AuNPs, Scheme 1).

**Scheme 1:**
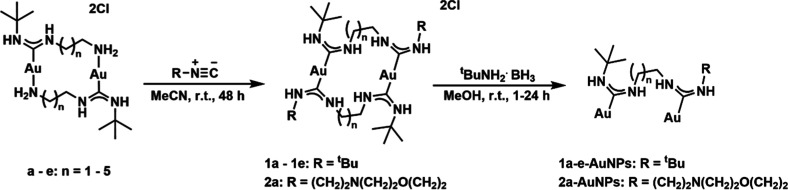
Synthesis Route to Form Complexes **1a**–**1e**,**2a** using Complexes **a**–**e** and Isocyanide Derivatives, before Further Reduction to **1a**-**e**- and **2a**–**AuNPs**

In addition, an asymmetric ADC Au­(I) complex (**2a**, Scheme 1) with a morpholine group was also synthesized to endow water-solubility to the resulting complex and ADC-AuNPs (**2a-AuNPs**). The synthesis of such asymmetric complexes is extremely challenging and only possible via the dimeric gold complexes (**a**–**e**). Typically, the reaction of isocyanide gold complex with diamines results in the formation of symmetric homodimers and thus yields symmetrically substituted ADC complexes.^41–44^ Therefore, this approach provides a novel, straightforward method for synthesizing a library of asymmetric gold complexes depending on the different isocyanide ligands used in each step.

## Results and Discussion

The addition of an isocyanide ligand to the solution of the aforementioned dimeric gold complexes (**a**–**e**, Scheme 1) resulted in its insertion into the gold amine bond, yielding dimeric gold complexes with different alkyl chain linkers (**1a**–**e**, Scheme 1). In the resulting structures, each gold atom is coordinated by two trans ADC ligands, with a total of four ADCs within the dimeric complex (**1a**–**e**, Scheme 1). In addition, to demonstrate the versatility of this method, an asymmetric tetrakis-ADC complex was formed by reacting a different isocyanide ligand with a dimeric gold complex, specifically, morpholinoethyl isocyanide was reacted with **a** to form the water-soluble complex, **2a** (Scheme 1).

The successfully prepared tetra-coordinated gold complexes (**1a–1e,2a**) were characterized by ^1^H and ^13^C NMR spectroscopy (Figures S1–S12). Due to the possible rotation along the N–C bond, the ^1^H NMR of ADC complexes can become complicated due to the formation of stereoisomers or rotamers in solution, with each rotamer contributing a set of signals, resulting in peak broadening. At room temperature, the rotation is low enough to be resolved in the NMR time scale; therefore, different isomers of each peak can be observed.^45^

Nevertheless, the new symmetries of the tetracarbene complexes were reflected by signals from the methylene proton resonances in the ^1^H NMR spectra. Formation of the trans carbene complexes also resulted in a downfield shift for the resonance of the carbene atom in the ^13^C NMR, for example, from δ 192.2 ppm in complex **d** to δ 207.2 ppm in complex **1d** (Figure S8).^41^ This downfield shift is characteristic of stronger trans ligands.^46^

The formation of the tetrakis-ADC gold complexes was also confirmed via high-resolution electrospray ionization mass spectrometry (HR-ESI-MS) by the presence of one distinct molecular mass peak from the [M-2Cl^–^]^2+^ species (see Figures S13–S18 in the ESI).

Furthermore, the structures of complexes **1a** and **1d** were examined using single-crystal X-ray diffraction (SC-XRD). The X-ray crystallographic analysis of compound **1d** revealed the expected bimetallic structure, as depicted in Figure 2. The asymmetric unit contained half a molecule of **1d**, with the second half generated by a 2-fold rotation axis. It was also revealed that the two Au–C_ADC_ bond lengths of the dimeric Au­(I) complex were slightly longer than the previously reported Au–C_ADC_ bond lengths (2.048(3) Å and 2.063(3) Å vs. 2.00–2.02 Å, respectively).^41^ The increase in bond length was attributed to the stronger σ donor strength of the carbenes located trans to each other compared to when chlorides or amines were trans to the carbene.^41^ A similar bimetallic structure with the expected shorter linker between the carbenes compared to **1d** was observed for compound **1a** (see Figures S19 and S20 in the ESI for detailed crystallographic data of compounds **1a** and **1d**).

**Figure 2:**
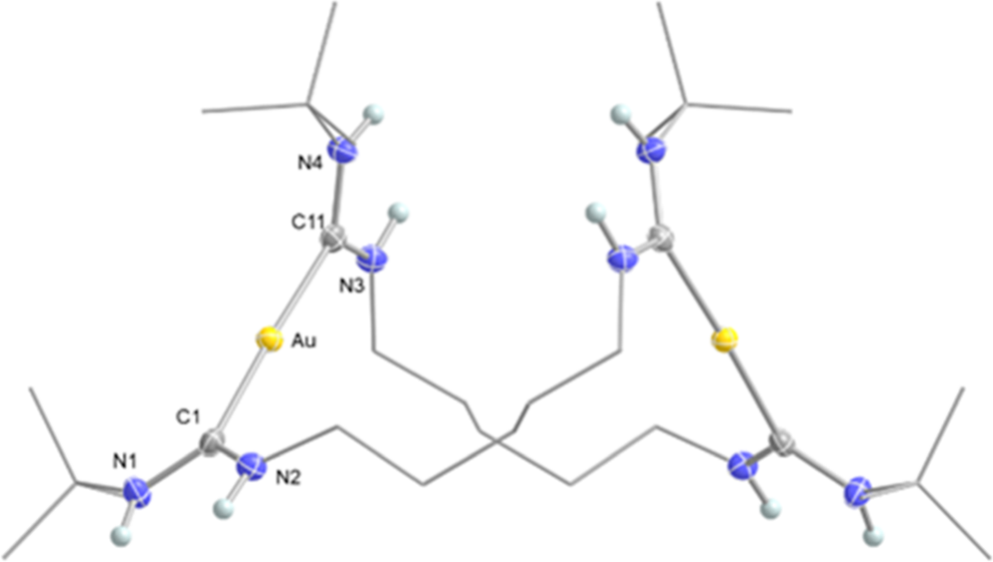
Molecular structure of **1d** (ellipsoids drawn at 50% probability, *t*-butyl groups and pentamethylene chain drawn as wireframe for clarity, hydrogen atoms except for those attached to nitrogen have been omitted). Selected bond lengths [Å] and angles [°]: Au–C1 2.046(3), Au–C11 2.053(3), C1–N1 1.336(3), C1–N21.337(4), C11–N3, 1.335(5), C11–N4 1.325(4); C1–Au–C11 177.3(1), N1–C1–N2 114.2(3), N3–C11–N4 114.5(3).

Having successfully found a reliable method to synthesize the Au complexes bearing bidentate ligands, the Au­(I) complexes were then reduced to form Au(0)­NPs, with the bidentate ligands stabilizing the surface of the particles. A proven method to obtain AuNPs is the reduction of gold­(I) complexes with ^*t*^BuNH_2_·BH_3_ (*tert*-butylamine borane).^47^ By reacting the complexes (compounds **1a–1e**,**2a**) with ^*t*^BuNH_2_·BH_3_ in methanol, a series of ADC-AuNPs was afforded (Scheme 1).

The formation of AuNPs could be observed visually by a change in the solution color to deep brown from colorless, which also indicated that the obtained AuNPs were around 2 nm^48^ with a very weak surface plasmon resonance (SPR) band, as can be seen in the UV–vis spectra (Figure S21). Additionally, **2a-AuNPs** showed a slightly larger size of *ca*. 4 nm; however, the corresponding SPR band remained relatively weak in intensity compared to NHC-stabilized AuNPs with a similar size.^49^ This could be due to the reduced resonance exhibited by the ADC ligand, as shown previously for NHC-stabilized quantum dots.^50^

The formation of gold nanoparticles was also verified by ^1^H and ^13^C NMR spectroscopy (Figures S22–S33); the former was used to confirm the retention of the ADC ligand, while the latter can be used to identify the presence of the carbene signal. For **1d-AuNPs**, the carbene peak was detected at δ 207.2 ppm (see Figure S29 in the ESI), indicating that the carbene–gold bond is still present after reduction.

Furthermore, X-ray photoelectron spectroscopy (XPS) was performed on **1a** and **1a-AuNPs** as representative examples to confirm the formation of the ADC on the gold and retention after AuNP formation. As expected, **1a** shows the presence of Au­(I) only with contributions at 88.8 and 85.2 eV for Au 4f_5/2_ and Au 4f_7/2_, respectively (Figure 3A). After reduction, **1a-AuNPs** show contributions from both Au­(I) and Au(0) (Figure 3B), with peaks at 87.0 and 83.4 eV for Au(0) 4f_5/2_ and Au 4f_7/2_, respectively. The presence of Au­(I) on the AuNP surface has been previously reported for bottom-up synthesized NHC stabilized AuNPs,^39,40,47,49^ indicating a similar mechanism during the reduction of molecular ADC-Au­(I) complexes to ADC-AuNPs. Additionally, the N 1s peaks at 400.0 eV (Figure 3C) and 399.7 eV (Figure 3D) can be assigned to the presence of the ADC–Au bond, as previously reported, with the absence of the isocyanide species at 401.7 eV.^51,52^ In comparison to NHC-AuNPs, the N 1s of the ADC–Au bond has a slightly lower binding energy (401 ^24^ vs. 399.7 eV, respectively), which can be attributed to the weaker π donation of the ADC nitrogen atoms vs. NHC nitrogen atoms. The C 1s peaks also provide structural information on the ADC-Au structure, with the absence of the isocyanide peak at 286.93 eV,^51,52^ but the presence of two peaks contributing to C–C/C–H and C–N bonds (Figure 3E: **1a**: 284.9 and 286.5 eV, respectively, and Figure 3F: **1a-AuNPs**: 284.8 and 286.4 eV, respectively). These values are in line with C 1s binding energies reported for bidentate-NHC-AuNPs.^40^

**Figure 3:**
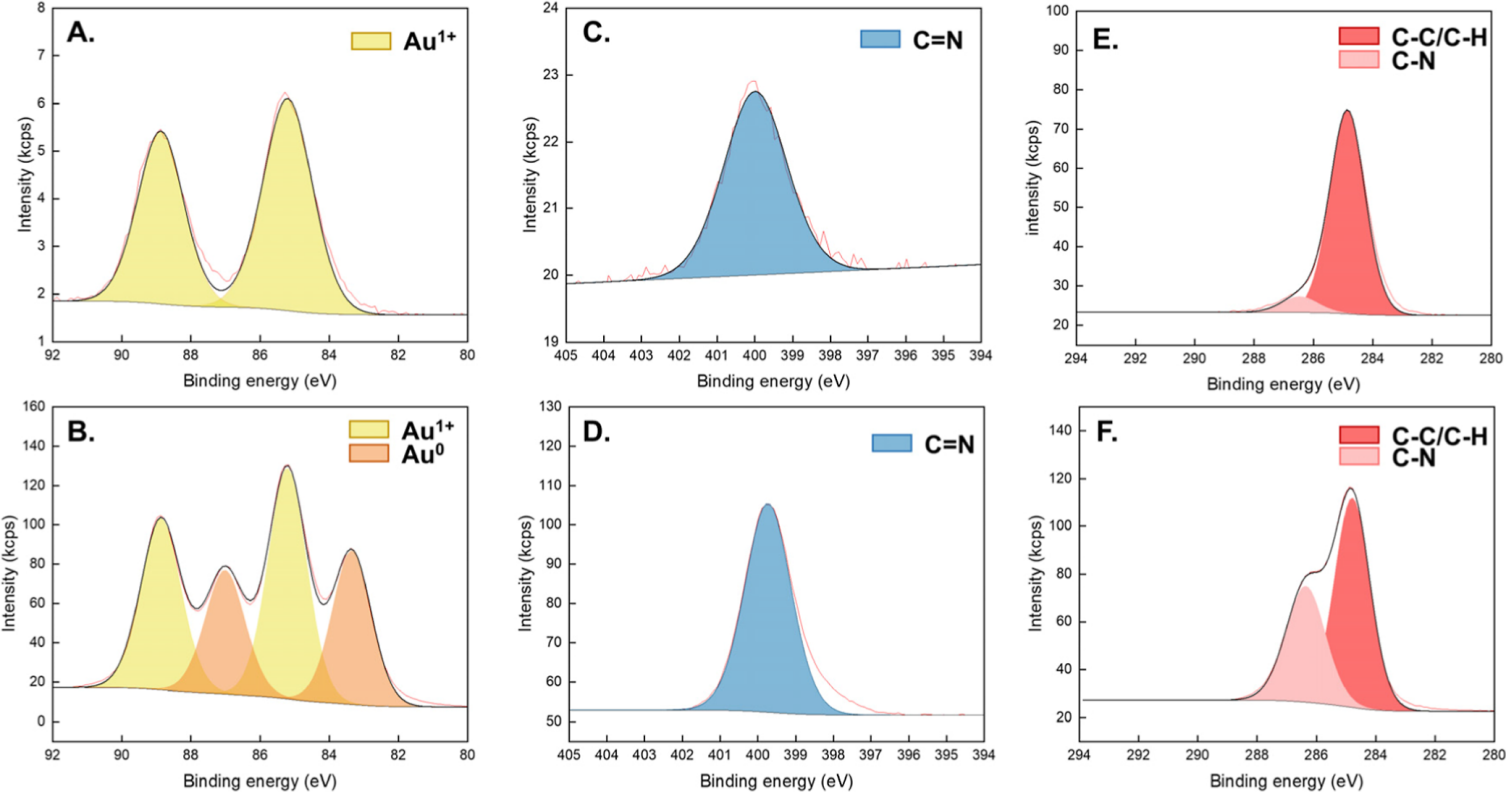
XPS spectra of Au 4f in (A). **1a** and (B). **1a-AuNPs**; N 1s in (C). **1a** and (D). **1a-AuNPs**; and C 1s in (E). **1a** and (F). **1a-AuNPs**.

The dispersity and size of the synthesized ADC-AuNPs were verified by TEM microscopy (Table 1, Figure 4A and S34–S38). Micrographs of sample films obtained via drop-casting a solution of **1a-AuNPs** in methanol and drying revealed the presence of spherical ADC-AuNPs with a bimodal size distribution with two average sizes of 1.38 ± 0.33 and 3.87 ± 0.63 nm (Figure S34). The other nanoparticles showed an average size distribution between 2 and 3 nm (Table 1 and Figures S35–S38), with the largest particle size observed for **2a-AuNPs** (4.01 ± 0.70 nm, Figure 4A).

**Table 1:** Average AuNP Size Measured by TEM Analysis

AuNPs	average size (nm)
1a-AuNPs	1.38 ± 0.33
	3.87 ± 0.63
1b-AuNPs	3.29 ± 1.01
1c-AuNPs	2.01 ± 0.53
1d-AuNPs	1.72 ± 0.43
1e-AuNPs	2.08 ± 0.63
2a-AuNPs	4.01 ± 0.70

**Figure 4:**
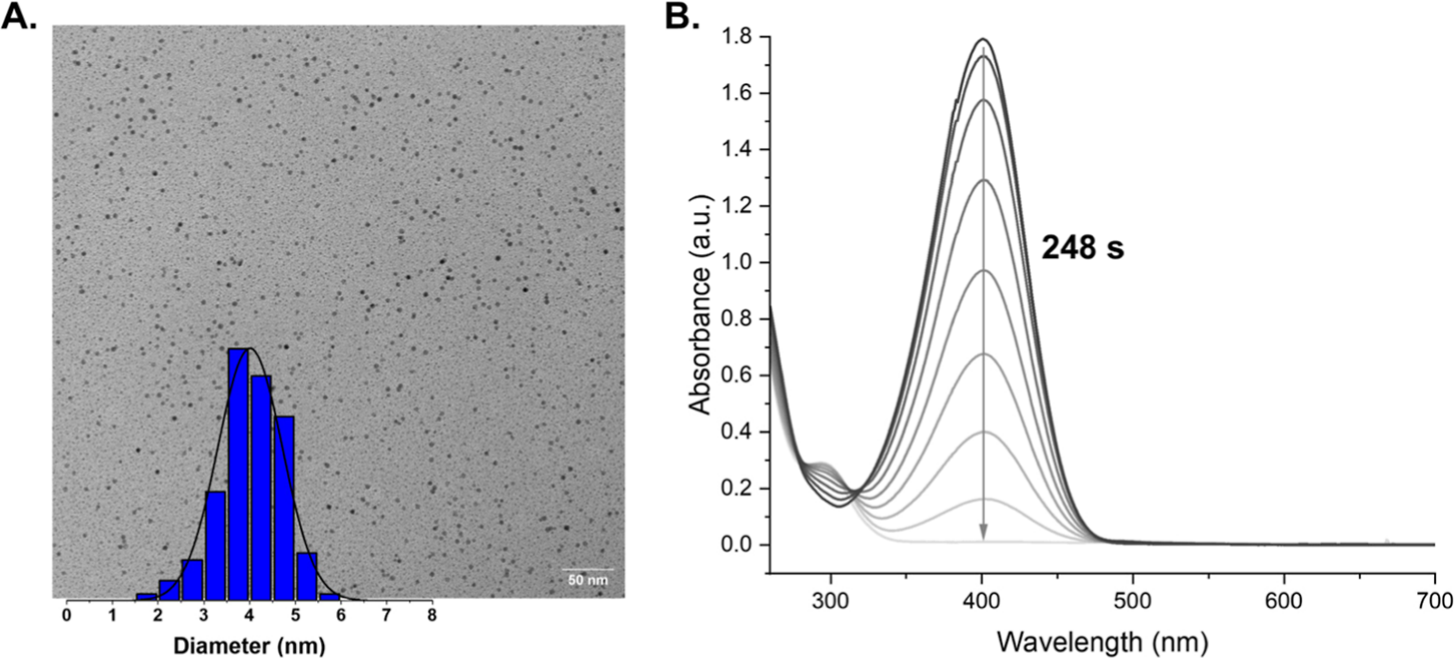
(A). TEM micrograph of **2a-AuNPs** with average size histogram. Average particle size measured = 4.01 ± 0.70 nm. (B). UV–vis absorption kinetic studies for the reduction of 4-nitrophenol (C_0_ = 0.05 mM) catalyzed by **2a-AuNPs** in H_2_O at room temperature.

Thermogravimetric analysis (TGA) was performed to estimate the amount of ADC ligands present on the AuNP surfaces. The estimated organic content was found to be similar for all NPs investigated, between 42 and 52% (see Figures S39–S44 in the ESI). These results are similar to those previously reported for bidentate NHC-AuNPs formed via “bottom-up” synthesis, with more ligand present on the BU-NPs compared to “top-down” synthesized NHC-AuNPs.^24,40^

Furthermore, to demonstrate the applicability of **2a-AuNPs** in more physiologically relevant conditions, its stability was tested in Milli-Q water over 24 h at room temperature by a UV–vis stability study (Figure S45). The **2a-AuNPs** showed good stability with a slight increase in absorbance and bathochromic shift over time, which is indicative of NP ripening. Next, **2a-AuNPs** were incubated with glutathione (2 mM), an intracellular reducing agent ubiquitous in the human body.^53^ Similarly, over 24 h, only a small loss in absorbance (*ca*. 20% decrease) was observed in the UV–vis spectra over time (Figure S46).

To further assess the stability of **2a-AuNPs** in physiologically relevant conditions, the NPs were suspended in phosphate-buffered saline (PBS 1×, pH 7.4, Figure S47A), aqueous solutions of pH 3 (Figure S47B) and pH 10 (Figure S47C), as well as in Milli-Q water at 50 °C (Figure S47D) over 48 h. The stability study in PBS 1× (Figure S47A) appeared similar to the data observed with Milli-Q water (Figure S45), with a small amount of NP ripening. At pH 3 (Figure S47B), there is a significant increase in absorbance over time, resulting in a stronger SPR band, most likely due to NP ripening. At pH 10 (Figure S47C), the increase in absorbance was even more significant, indicating a lower stability at a more basic pH. Finally, the stability of **2a-AuNPs** in Milli-Q water at 50 °C showed the lowest stability overall, with a significant increase in absorbance up to *ca*. 34 h (arrow 1, Figure S47D) before a decrease in absorbance up to 48 h (arrow 2, Figure S47D) due to the aggregation of AuNPs, which could also be seen by the formation of a black precipitate. Overall, while the stability of these systems requires further optimization, this study provides a first proof-of-concept for their synthetic feasibility. With continued refinement, we anticipate that a stability profile comparable to NHC–AuNPs can be achieved.

Following this, to correlate the ligand properties with function, the catalytic activity of the ADC-AuNPs was investigated using a model system, the reduction of 4-nitrophenol (4-NP) to 4-aminophenol (4-AP) with an excess amount of sodium borohydride (NaBH_4_) in water. For AuNPs **1a**–**e**, methanol was required (0.5%) to dissolve the NPs. The activity was monitored by measuring the time-dependent adsorption spectra of the reaction mixture solution. In the absence of AuNPs, the mixtures of 4-NP and NaBH_4_ show a strong adsorption peak at ca. 400 nm due to the 4-nitrophenolate species (Figure S48).

From the UV–vis graphs, the conversion and rate constant could be determined by assuming a *pseudo*-first-order reaction. A conversion of >99% was obtained using **1a-AuNPs**, **1b-AuNPs**, **1c-AuNPs**, and **2a-AuNPs** as catalysts, while for **1d-AuNPs** and **1e-AuNPs**, a conversion of only 42% and 44% was measured, respectively (Figures 4B and S49A–53A). The rate constants for **1a-AuNPs**, **1b-AuNPs**, **1c-AuNPs** and **2a-AuNPs** were calculated to be *k* = 0.00613 s^–1^ (0.3678 min^–1^), *k* = 0.00634 s^–1^ (0.3804 min^–1^), *k* = 0.00241 s^–1^ (0.1446 min^–1^) and *k* = 0.0091 s^–1^ (0.546 min^–1^), respectively (Figures S49–53B,54). To compare the catalytic activity of these ADC-AuNPs with NHC-AuNPs in the literature for the reduction of 4-NP, the rate constants can be normalized (*k*_nor_ = *k*/mass of catalyst).^54,55^ In comparison to another bidentate NHC–AuNP system, most of the ADC-AuNPs in this study outperform it, with only **1c-AuNPs** showing comparable activity (**1c-AuNPs** = 0.723 min^–1^ mg^–1^ vs. NHC-AuNPs = 0.7 min^–1^ mg^–1^).^40^ However, when compared to the monodentate system from the same study (7 min^–1^ mg^–1^)^40^ the ADC-AuNPs show lower overall activity; therefore, there must always be a balance between stability and catalytic activity when comparing monodentate and bidentate ligands.

Overall, the longer the alkyl linker between the gold centers, the less catalytically active the ADC-AuNPs are. This could be due to the increased steric effect of the longer alkyl chains or the increased hydrophobicity of the ligand, and since the 4-nitrophenolate anion is hydrophilic, this could reduce the amount of substrate reaching the AuNP surface. Furthermore, to assess whether the activity of the AuNPs was due to the Au­(I) species, we performed the reduction of 4-nitrophenol with the gold complex, **2a**, in the presence of excess NaBH_4_. Due to the presence of a reducing agent, the Au complex is expected to be reduced to AuNPs; however, in the first few minutes, no conversion of 4-nitrophenol was observed, with only a small amount of conversion observed over 10 min (Figure S55).

## Conclusions

In conclusion, a series of gold nanoparticles stabilized by acyclic diamino carbenes were synthesized, including an asymmetric water-soluble derivative. The ADC system allowed the formation of AuNPs stabilized in a bidentate fashion, with the water-soluble derivative showing high stability in physiologically relevant conditions. The AuNPs also demonstrated catalytic activity, which was dependent on the linker length between the ADCs. This study grants facile access to asymmetric substituted chelating ADCs, allowing the fine-tuning of gold nanoparticles for catalytic applications. Future work will involve the modification of these NPs with more asymmetric ligands for different functions, for example, the addition of fluorophores for cellular tracking of the NPs. The linker chain can also be varied to endow the NP with different reactivity and properties, for example, installation of a chiral linker to produce chiral NPs.

## Experimental Section

### Materials and Methods

All experiments were performed under ambient conditions unless stated otherwise. Borane *tert*-butylamine complex and *n*-pentane were purchased from Sigma-Aldrich, 2-morpholinoethyl isocyanide was purchased from Aldrich, *tert*-butyl isocyanide was purchased from Alfa Aesar, and dichloromethane, diethyl ether, and *n*-hexane were purchased from VWR chemicals. All purchased chemicals were used as received. Dimeric *n*-acyclic carbene gold complexes (**a**–**e**) were synthesized according to a literature procedure.^41^ No uncommon hazards are noted.

^1^H and ^13^C NMR spectra were recorded at the NMR Centre, Faculty of Chemistry, University of Vienna, utilizing a Bruker 600 MHz spectrometer, with TMS δ *H* = 0 or residual protic solvent peak [MeOD, δ *H* = 3.31] as the internal standard. Chemical shifts are given in ppm (δ).

Ultraviolet–visible (UV–vis) spectroscopy was carried out using a PerkinElmer spectrophotometer Lambda 35. Samples were prepared at a concentration of 100 μg/mL in water/methanol (200/1), unless otherwise stated. Stability studies of **2a-AuNPs** were performed with a concentration of 250 μg/mL in Milli-Q H_2_O at room temperature and 50 °C, or aqueous GSH (2 mM) at room temperature. The stability studies at room temperature were monitored over 24 h and studies at 50 °C were monitored over 48 h with a measurement every hour.

Additional stability studies of **2a-AuNPs** were performed using the Tecan Spark Plate Reader. The samples were prepared as stock solutions by suspending **2a-AuNPs** in 1× PBS, pH 3 aqueous solution, or pH 9 aqueous solution with a concentration of 250 μg/mL. In a 96-well plate (Sarstedt, ELISA plate, 96 well, flat base, PS, white, High Binding), five wells were filled with 300 μL of either the blank solution or the AuNP-containing solutions. The experiment was performed over 48 h, by taking measurements every hour, with each well run in quintuplicate. The final absorbance was then calculated by subtracting the absorbance of the blank solutions from that of the samples. Subsequently, an average absorbance over five wells was obtained and reported. The pH 3 and pH 9 aqueous solutions were prepared by adding hydrochloric acid (Sigma-Aldrich, 37%) or sodium hydroxide (Sigma-Aldrich, ≥98% pellets), respectively, in Milli-Q H_2_O. The pH was measured using a pH meter and adjusted with HCl or NaOH. The PBS 1× solution was prepared from a PBS 10× solution by mixing 9:1 Milli-Q H_2_O: PBS 10× (Alfa Aesar). The final solution was used without further modifications.

The average diameter (D) and the size distribution of the nanoparticles were determined using ImageJ software and by measuring 100 randomly selected nanoparticles in arbitrarily chosen areas of various obtained images. The size distribution is reported as the standard deviation (σ), which is calculated according to the following formula: σ = {(Di–D)^2^/(*n*–1)}^1/2^.

High-resolution electrospray ionization mass spectrometry (HR-ESI-MS) was performed at the Mass Spectrometry Centre, Faculty of Chemistry, University of Vienna, utilizing a Bruker maXis UHR-TOF or Thermo Orbitrap Exploris MS.

Single crystal X-ray diffraction data were collected with a Stadivari Diffractometer (STOE & Cie GmbH, Germany) equipped with an EIGER2 R500 detector (Dectris Ltd., Switzerland). Data were processed and scaled with the STOE software suite X-Area (STOE & Cie GmbH). Structures were solved with SHELXT^56^ and refined with SHELXL.^57^ The structures were validated with CHECKCIF (https://checkcif.iucr.org/). See the respective CIF file for exact versions and more details.

X-ray photoelectron spectroscopy (XPS) was performed on a Nexsa photoelectron spectrometer (Thermo Fisher Scientific, UK) by the Core Facility “interface characterization”, Faculty of Chemistry, University of Vienna. Samples were drop-cast from solutions in methanol onto a freshly cleaned silicon wafer and dried at 70 °C. The silicon wafers were cleaned by sonication in methanol and water before drying. All measurements were performed using Al–Kα X-ray radiation at 72 W and a pass energy of 200 eV with a spot size of 400 μm; a flood gun was used to eliminate charge buildup. The high-resolution spectra (step size of 0.1 eV) of the single elements were acquired with 50 passes at pass energies of 50 eV. Obtained spectra were evaluated using the Advantage software package v5.9929 provided by Thermo Fisher Scientific and Origin Pro v9.6.0.172 using Shirley Background. Further data can be found in Table S1.

TEM solid samples were dispersed in 100% methanol. Five μL drops of all samples were put onto carbon-coated copper grids and allowed to dry in an oven at 70 °C. Images were obtained at the Electron Microscopy Facility, Institute of Science and Technology Austria, using a TVIPS EM-Menu software with camera OSIS Megaview G3 attached to a Tecnai 10 TEM running at 160 kV.

Thermogravimetric analysis (TGA) was performed using a Netzsch STA 449-F3 Jupiter or a Mettler-Toledo TGA/DSC 3+ instrument in the temperature range of 25–700 °C under N_2_ atmosphere (5 mL·min^–1^), at a heating rate of 10 °C min^–1^.

### Synthesis of ADC Tetramer Dimeric Gold Complexes

#### General Procedure

A previously reported NAC dimeric gold complex^41^ (compounds **a**–**e**, 1 equiv., 100 mg) was dissolved in 2 mL acetonitrile, and 2.5 equiv of ligand (*tert*-butyl isocyanide (**1a**–**e**) or morpholinoethyl isocyanide (**2a**)) was then added to the reaction mixture. The reaction mixture was stirred for 24 h at 22 °C. Subsequently, the reaction mixture was concentrated under reduced pressure, and 2 mL of cold *n*-pentane was then added. A filtration was performed, followed by washing three times with *n*-pentane. The obtained white precipitate in all cases was dried under vacuum overnight and used without further purification.

##### Complex **1a**

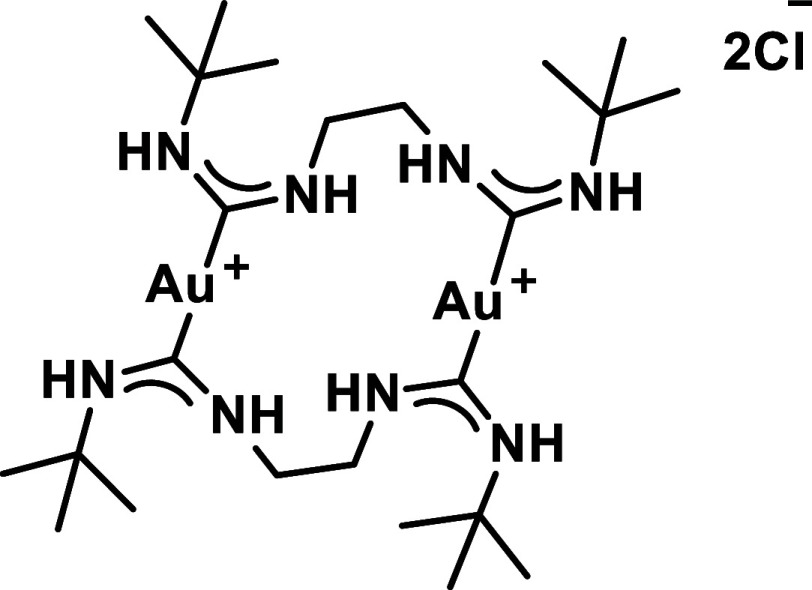



Yield: 86.6 mg (71%). ^1^H NMR (600 MHz, CD_3_OD): δ = 1.36–1.61 (36H, CH_3_); 3.44–3.49 (2H, CH_2_); 3.88–4.02 (6H, CH_2_). ^13^C NMR (150 MHz, CD_3_OD): δ = 29.32 (CH_3_); 32.07 (CH_3_); 43.85 (CH_2_); 51.32 (CH_2_); 53.98 (*C*(CH_3_)_3_); 207.95 ((NH)­(NH)*C*Au) ppm. HR-ESI-MS (*m*/*z*): Calcd for [C_24_H_52_Au_2_N_8_]^2+^: 423.18. Found [C_24_H_52_Au_2_N_8_]^2+^: 423.23. Anal. Calcd for C_24_H_52_N_8_Au_2_Cl_2_·2H_2_O: C, 30.23; H, 5.92; N, 11.75. Found: C, 29.96; H, 5.71; N, 12.14.

##### Complex **1b**

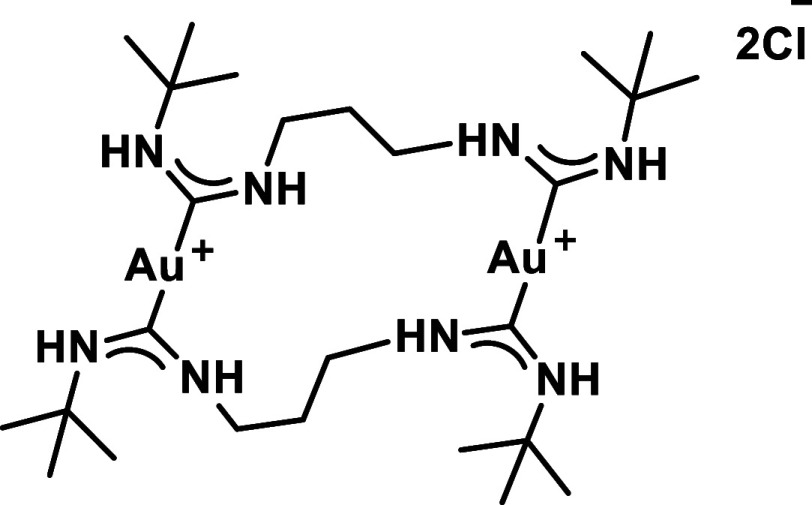



Yield: 96.5 mg (80%). ^1^H NMR (600 MHz, CD_3_OD): δ = 1.39–1.61 (36H, CH_3_); 1.95–2.02 (4H, CH_2_); 3.25 (2H, CH_2_); 3.69–3.74 (6H, CH_2_). ^13^C NMR (150 MHz, CD_3_OD): δ = 28.81 (CH_3_); 31.93 (CH_3_); 33.95 (CH_2_); 39.73 (CH_2_); 48.06 (CH_2_); 53.61 (*C*(CH_3_)_3_); 207.22 ((NH)­(NH)*C*Au) ppm. MS (*m*/*z*): calcd for [C_26_H_56_N_8_Au_2_Cl]^+^: 909.3642; found [C_26_H_56_N_8_Au_2_Cl]+: 909.3646 and [C_26_H_56_N_8_Au_2_]^2+^: 437.1992. Anal. Calcd for C_26_H_56_N_8_Au_2_Cl_2_·2H_2_O: C, 31.81; H, 6.16; N, 11.41. Found: C, 31.69; H, 6.00; N, 11.80.

##### Complex **1c**

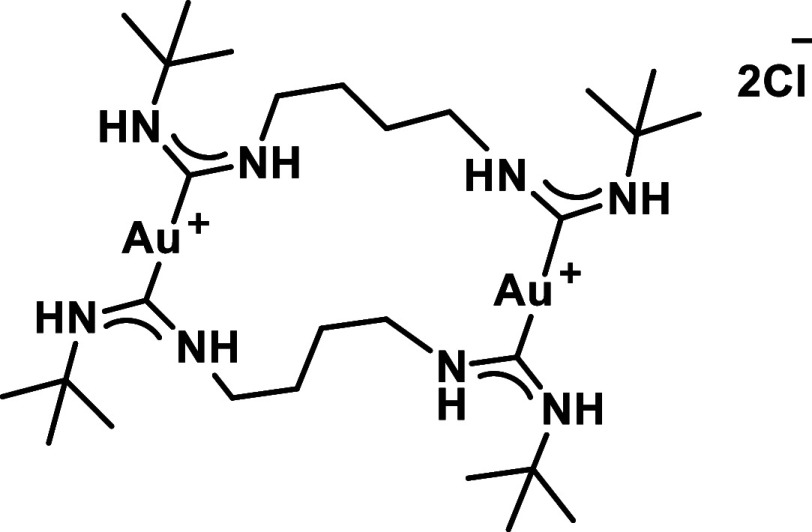



Yield: 42.9 mg (36%). ^1^H NMR (600 MHz, CD_3_OD): δ = 1.39–1.60 (36H, CH_3_); 1.67–1.70 (8H, CH_2_); 3.19 (2H, CH_2_); 3.66–3.70 (6H, CH_2_). ^13^C NMR (150 MHz, CD_3_OD): δ = 26.23 (CH_2_); 29.14 (CH_3_); 29.52 (CH_2_); 32.01 (CH_3_); 42.25 (CH_2_); 50.54 (CH_2_); 53.65 (*C*(CH_3_)_3_); 207.18 ((NH)­(NH)*C*Au) ppm. MS (*m*/*z*): calcd for [C_28_H_60_N_8_Au_2_Cl]+: 937.3955; found [C_28_H_60_N_8_Au_2_Cl]^+^: 937.3952 and [C_28_H_60_N_8_Au_2_]^2+^: 451.2139. Anal. Calcd for C_28_H_60_N_8_Au_2_Cl_2_·2H_2_O: C, 33.31; H, 6.39; N, 11.10. Found: C, 33.09; H, 6.14; N, 11.47.

##### Complex **1d**

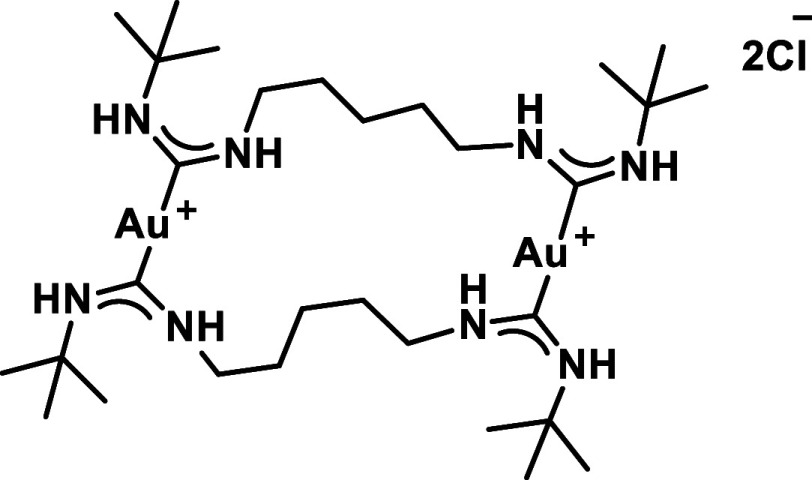



Yield: 20.8 mg (17%). ^1^H NMR (600 MHz, CD_3_OD): δ = 1.36 (2H, CH_3_); 1.39–1.40 (4H, CH_2_); 1.54–1.60 (34H, CH_3_); 1.65–1.66 (8H, CH_2_); 3.16 (2H, CH_2_); 3.61–3.66 (6H, CH_2_). ^13^C NMR (150 MHz, CD_3_OD): δ = 24.74 (CH_2_); 28.72 (CH_2_); 29.13 (CH_3_); 30.12 (CH_3_); 31.80 (CH_3_); 32.30 (CH_2_); 42.46 (CH_2_); 50.59 (CH_2_); 53.61 (*C*(CH_3_)_3_); 207.20 ((NH)­(NH)*C*Au) ppm. MS (*m*/*z*): calcd for [C_30_H_64_N_8_Au_2_Cl]+: 965.4268; found [C_30_H_64_N_8_Au_2_Cl]^+^: 965.4258 and [C_30_H_64_N_8_Au_2_]^2+^: 465.2295. Anal. Calcd for C_30_H_64_N_8_Au_2_Cl_2_·2H_2_O: C, 35.33; H, 6.52; N, 10.99. Found: C, 35.29; H, 6.45; N, 10.89.

##### Complex **1e**

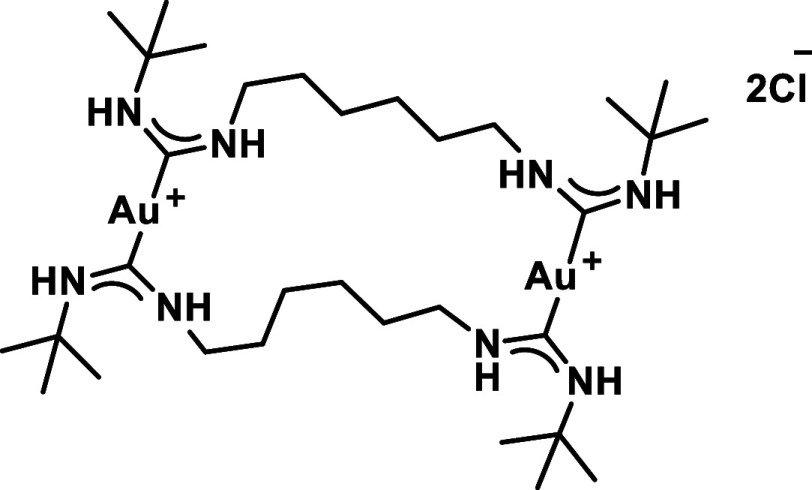



Yield: 30.6 mg (25%). ^1^H NMR (600 MHz, CD3OD): δ = 1.37 (2H, CH_3_); 1.39–1.40 (8H, CH_2_); 1.54–1.60 (34H, CH_3_); 1.62–1.64 (8H, CH_2_); 3.15 (2H, CH_2_); 3.61–3.65 (6H, CH_2_). ^13^C NMR (150 MHz, CD_3_OD): δ = 27.45 (CH_2_); 28.91 (CH_2_); 29.88 (CH_3_); 31.94 (CH_3_); 32.38 (CH_2_); 42.45 (CH_2_); 50.69 (CH_2_); 53.56 (*C*(CH_3_)_3_); 207.12 ((NH)­(NH)*C*Au) ppm. MS (*m*/*z*): calcd for [C_32_H_68_N_8_Au_2_]^2+^: 479.25; found C_32_H_68_N_8_Au_2_]^2+^: 479.2447. Anal. Calcd for C_32_H_68_N_8_Au_2_Cl_2_·H_2_O: C, 36.68; H, 6.73; N, 10.69. Found: C, 36.35; H, 6.68; N, 10.39.

##### Complex **2a**

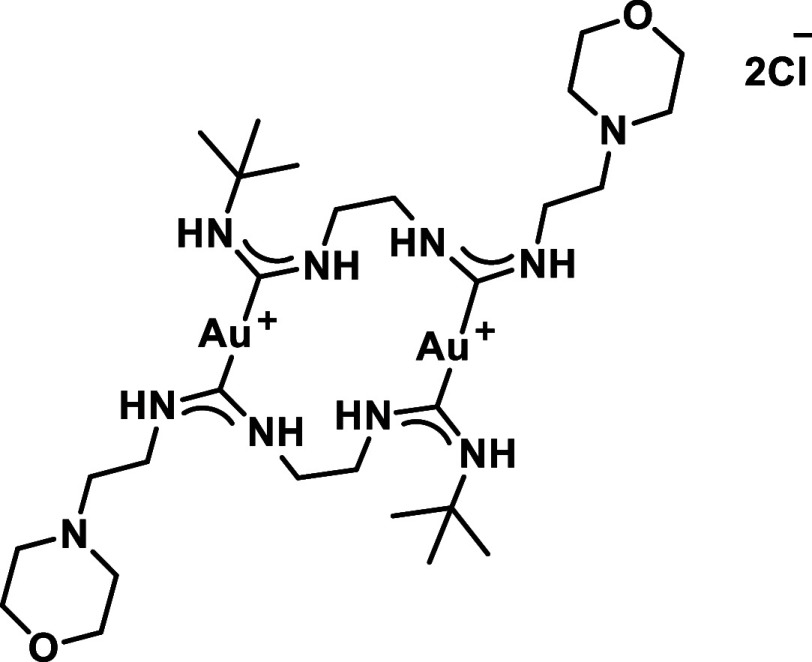



Yield: 98.3 mg (71%). ^1^H NMR (600 MHz, D_2_O): δ = 1.36–1.56 (18H, CH_3_), 2.58–2.68 (16H, CH_2_), 3.75–3.76 (16H, CH_2_). ^13^C NMR (151 MHz, CD_3_OD): δ = 31.82 (CH_3_), 32.23 (CH_3_), 39.88 (CH_2_), 46.23 (CH_2_), 53.84 (*C*(CH_3_)_3_), 56.45 (CH_2_), 59.44 (CH_2_), 66.96 (CH_2_), 207.50 ((NH)­(NH)*C*Au) ppm. Anal. Calcd for C_28_H_58_Au_2_Cl_2_N_10_O_2_·2H_2_O: C, 31.50; H, 5.85; N, 13.12. Found: C, 31.69; H, 5.85; N, 13.11.

### Synthesis of ADC Gold Nanoparticles (ADC-AuNPs)

#### General Procedure

A tetramer complex (**1a**–**e**, **2a**, 1 equiv, 100 mg) was dissolved in 2 mL methanol, and 2.5 equiv of *tert*-butylamine borane complex were then added to the reaction mixture. The reaction mixture was stirred for 24 h (**1a**, **2a**), 16 h (**1b**), 10 h (**1c**, **1d**), and 1 h (**1e**) at 22 °C since these were the times that would yield nanoparticles with similar size. Subsequently, a drop of water was added to the reaction mixture, followed by the addition of diethyl ether, which facilitated the centrifugation of the nanoparticles. The nanoparticles were subsequently washed and centrifuged several times using 1:100 MeOH/Et_2_O solvent mixture. Finally, the obtained particles were dried under vacuum and stored at 22 °C without any signs of decomposition and used for analysis and as catalysts without further purification.

##### 1a-AuNPs

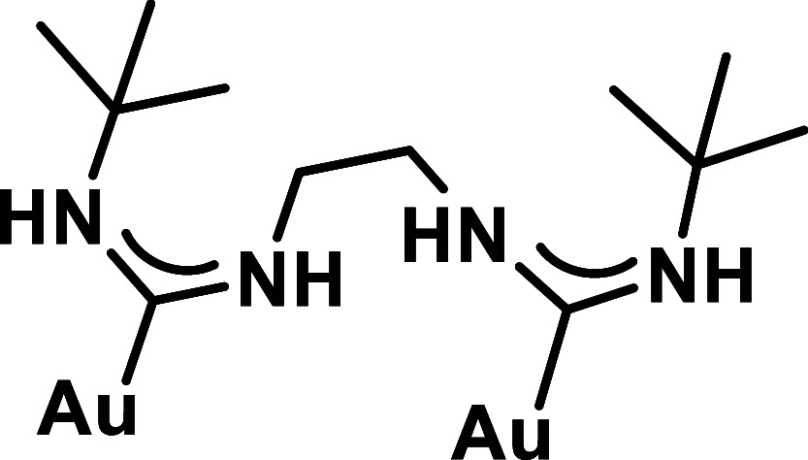



^1^H NMR (600 MHz, CD_3_OD): δ = 1.40–1.61 (18H, CH_3_); 3.42–3.43 (1H, CH_2_); 3.88 (3H, CH_2_). ^13^C NMR (150 MHz, CD_3_OD): δ = 29.04 (CH_3_); 32.07 (CH_3_); 43.86 (CH_2_) 51.33 (*C*(CH_3_)_3_); 53.99 (*C*(CH_3_)_3_); 207.96 ((NH)­(NH)*C*Au) ppm.

##### 1b-AuNPs

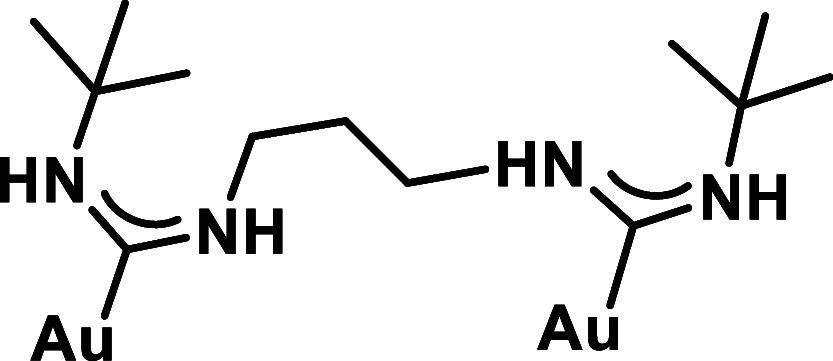



^1^H NMR (600 MHz, CD_3_OD): δ = 1.41–1.61 (18H, CH_3_); 1.94–1.98 (2H, CH_2_); 3.25 (1H, CH_2_) 3.70–3.71 (3H, CH_2_). ^13^C NMR (150 MHz, CD_3_OD): δ = 29.44 (CH_3_); 32.06 (CH_3_); 33.92 (CH_2_); 39.92 (CH_2_); 44.92 ((*C*(CH_3_)_3_); 53.78 (*C*(CH_3_)_3_); 207.39 ((NH)­(NH)*C*Au) ppm.

##### 1c-AuNPs

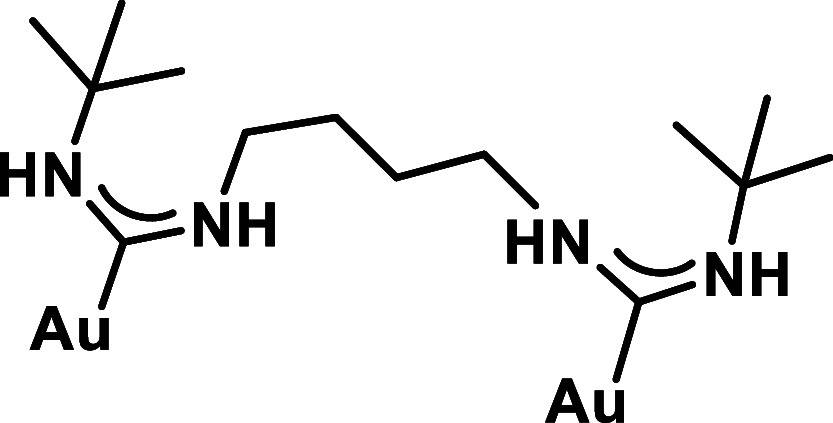



^1^H NMR (600 MHz, CD_3_OD): δ = 1.40–1.60 (18H, CH_3_); 1.66–1.70 (4H, CH_2_); 3.18–3.20 (1H, CH_2_); 3.65–3.69 (3H, CH_2_). ^13^C NMR (150 MHz, CD_3_OD): δ = 29.13 (CH_3_); 29.52 (CH_2_); 32.00 (CH_3_); 42.15 (CH_2_); 50.54 (*C*(CH_3_)_3_); 54.46 (*C*(CH_3_)_3_); 207.18 ((NH)­(NH)*C*Au) ppm.

##### 1d-AuNPs

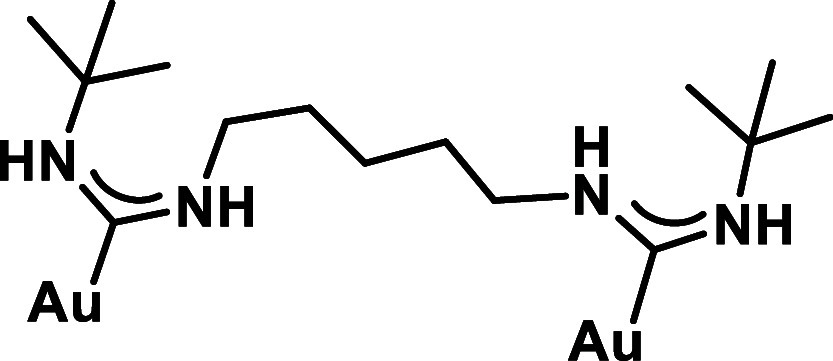



^1^H NMR (600 MHz, CD_3_OD): δ = 1.40–1.46 (4H, CH_2_); 1.54–1.60 (18H, CH_3_); 1.63–1.66 (4H, CH_2_); 3.15–3.17 (1H, CH_2_); 3.62–3.66 (3H, CH_2_). ^13^C NMR (150 MHz, CD_3_OD): δ = 28.72 (CH_2_); 29.13 (CH_3_); 31.93 (CH_3_); 32.15 (CH_2_); 42.46 (CH_2_); 50.60 ((*C*(CH_3_)_3_); 53.61 (*C*(CH_3_)_3_); 207.19 ((NH)­(NH)*C*Au) ppm.

##### 1e-AuNPs

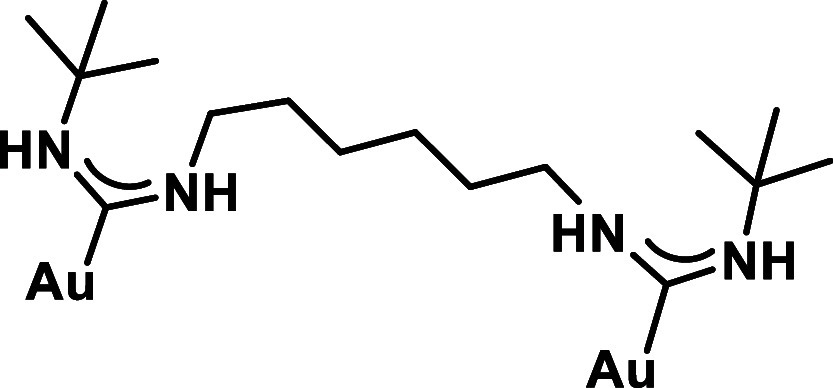



^1^H NMR (600 MHz, CD_3_OD): δ = 1.40 (2H, CH_2_); 1.42–1.46 (2H, CH_3_); 1.54–1.60 (16H, CH_3_); 1.66 (4H, CH_2_); 3.16 (1H, CH_2_); 3.62–3.66 (3H, CH_2_). ^13^C NMR (150 MHz, CD_3_OD): δ = 27.4 (CH_2_); 28.8 (CH_2_); 29.1 (CH_3_); 31.9 (CH_3_); 50.7 (CH_2_); 53.6 (*C*(CH_3_)_3_); 54.1 (*C*(CH_3_)_3_); 207.2 ((NH)­(NH)*C*Au) ppm.

##### 2a-AuNPs

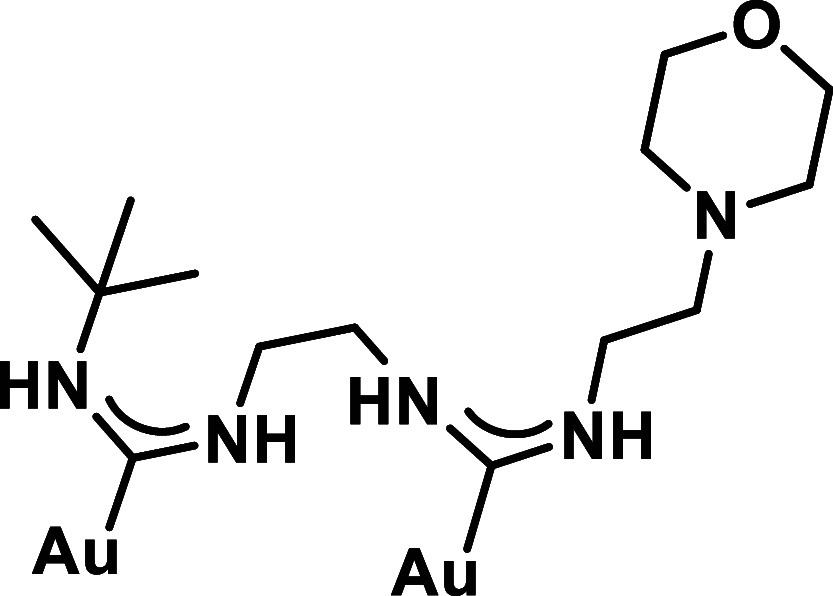



^1^H NMR (600 MHz, CD_3_OD): δ = 1.62–1.39 (18H, CH_3_), 2.66–2.52 (16H, CH_2_), 3.70–3.69 (16H, CH_2_). ^13^C NMR (151 MHz, CD_3_OD): δ = 31.92 (CH_3_), 32.21 (CH_3_), 41.64 (CH_2_), 46.88 (CH_2_), 54.72 (*C*(CH_3_)_3_), 57.67 (CH_2_), 60.38 (CH_2_), 67.80 (CH_2_), 208.75 ((NH)­(NH)*C*Au) ppm.

### Catalytic Reduction of 4-Nitrophenol with ADC-AuNPs

#### General Procedure

One mL of a 0.1 mM 4-nitrophenol aqueous solution was added to a quartz cuvette. To this solution, 0.20 mg of AuNPs in 10 μL methanol was added, and an additional 657 μL of water was added. In the case of **2a-AuNPs**, only Milli-Q water was used (667 μL). To initiate the reaction, 333 μL of a 30 mM NaBH_4_ solution was added. The measurements were performed directly from the quartz cuvette. The reaction was followed until completion or when no more reactivity was detected at room temperature.

In the control reaction, 0.20 mg of **2a** was used and the reaction was monitored over 10 min.

## Supplementary Material



## Data Availability

A previous version of this manuscript has been deposited on the preprint server ChemRxiv (10.26434/chemrxiv-2025-p23nb)^58^

## Notes

Additional characterization data: ^1^H NMR, ^13^C NMR, HR-ESI-MS of all compounds; supplementary X-ray crystallography data; UV–vis spectra, ^1^H NMR, ^13^C NMR, TEM, and TGA data for all AuNPs; stability studies for **2a-AuNPs**; results for catalytic studies (PDF)
