# Supplementary material for: Bidentate Acyclic Diamino Carbene-Stabilized Gold Nanoparticles from Symmetric and Asymmetric Gold(I) Complexes: Synthesis, Characterization, and Catalytic Activity
Source: Inorg Chem. 2025 Sep 12;64(38):19316–24. doi: 10.1021/acs.inorgchem.5c03050 (PMC12486202; doi:10.1021/acs.inorgchem.5c03050)
Supplement: Supplementary file 1 [file ic5c03050_si_001.pdf]

Supporting Information

Bidentate Acyclic Diamino Carbene-Stabilized Gold Nanoparticles from Symmetric and Asymmetric Gold(I) Complexes: Synthesis, Characterization, and Catalytic Activity

Sophie R. Thomas,[a] Tristan T. Y. Tan,[b] Guilherme M. D. M. Rubio,[a] Monnaya Chalermnon,[a] Jia Min Chin,[c*] and Michael R. Reithofer [a*]

[a] *Institute of Inorganic Chemistry, Faculty of Chemistry, University of Vienna, Währinger Str. 42, 1090 Vienna, Austria. Email: michael.reithofer@univie.ac.at*

[b] *Institute of Material Research and Engineering, A*STAR (Agency for Science, Technology and Research), 138634, Singapore.*

[c] *Department of Functional Materials and Catalysis, Faculty of Chemistry, University of Vienna, Währinger Str. 42, 1090 Vienna, Austria. Email: jiamin.chin@univie.ac.at*

^{1}H and ^{13}C NMR spectroscopy of gold complexes (1a-e, 2a)

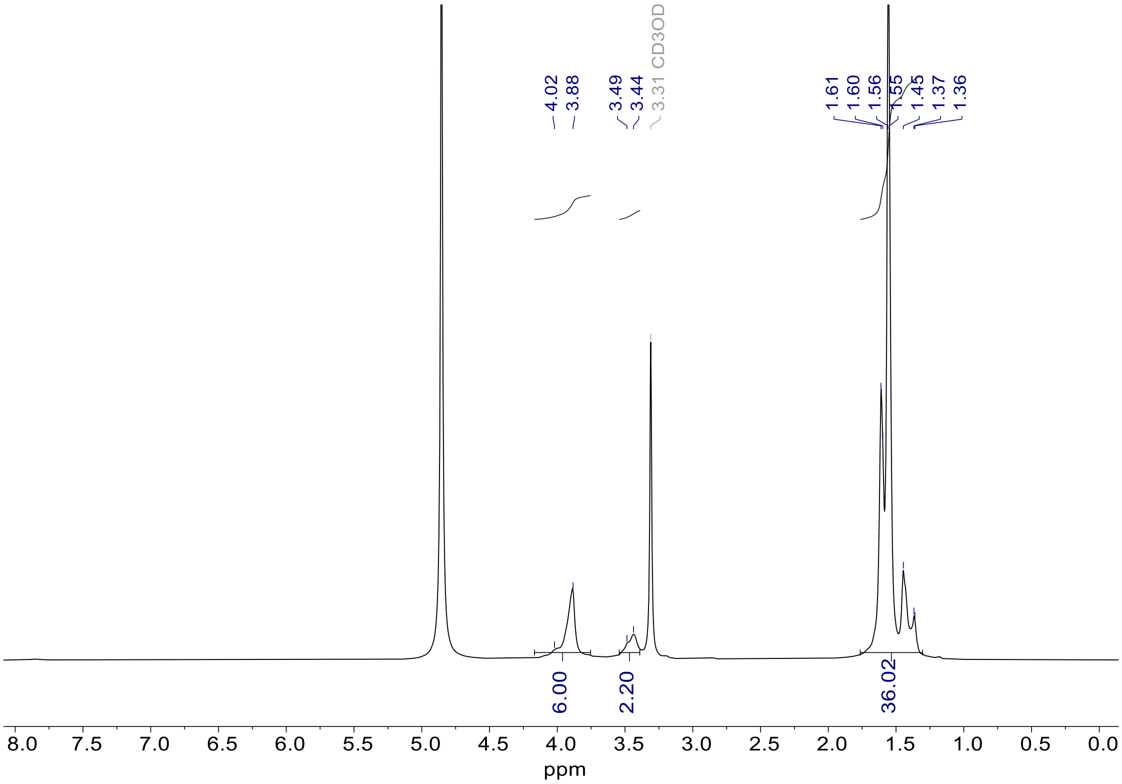

Figure S1. *^{1}H-NMR spectrum of complex **1a** in CD_3OD.*

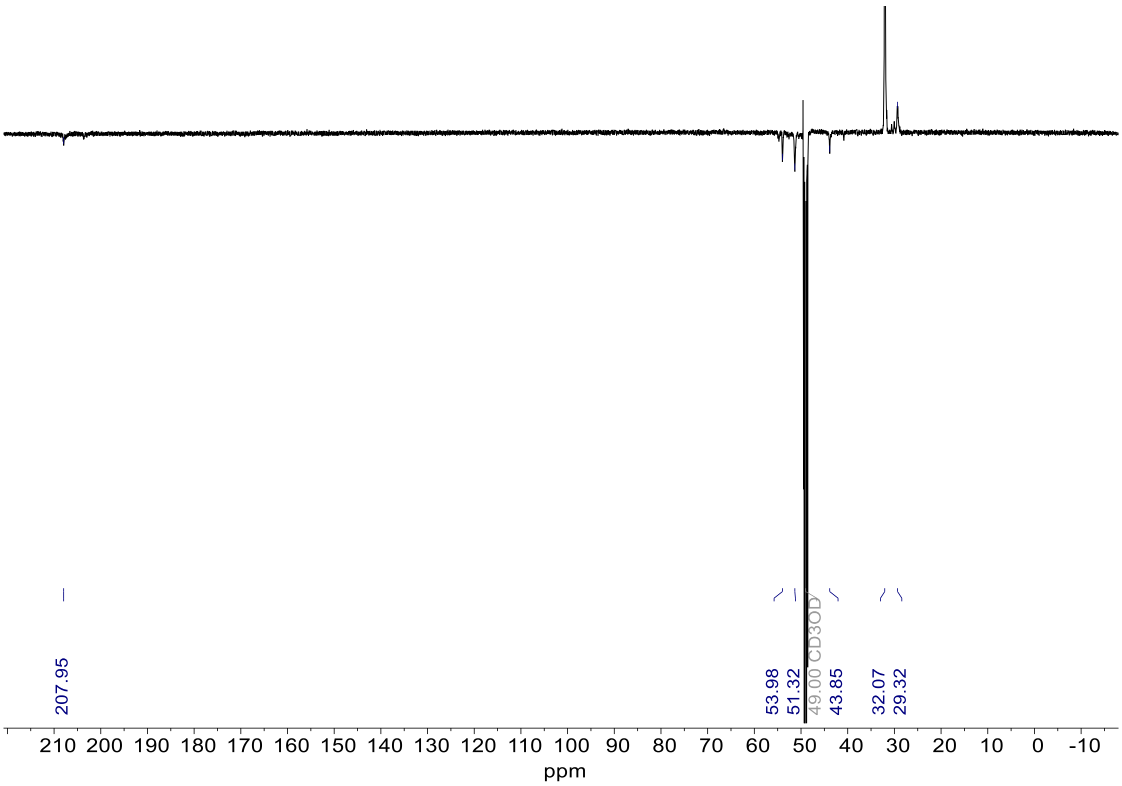

Figure S2. *^{13}C-NMR spectrum of complex **1a** in CD_3OD.*

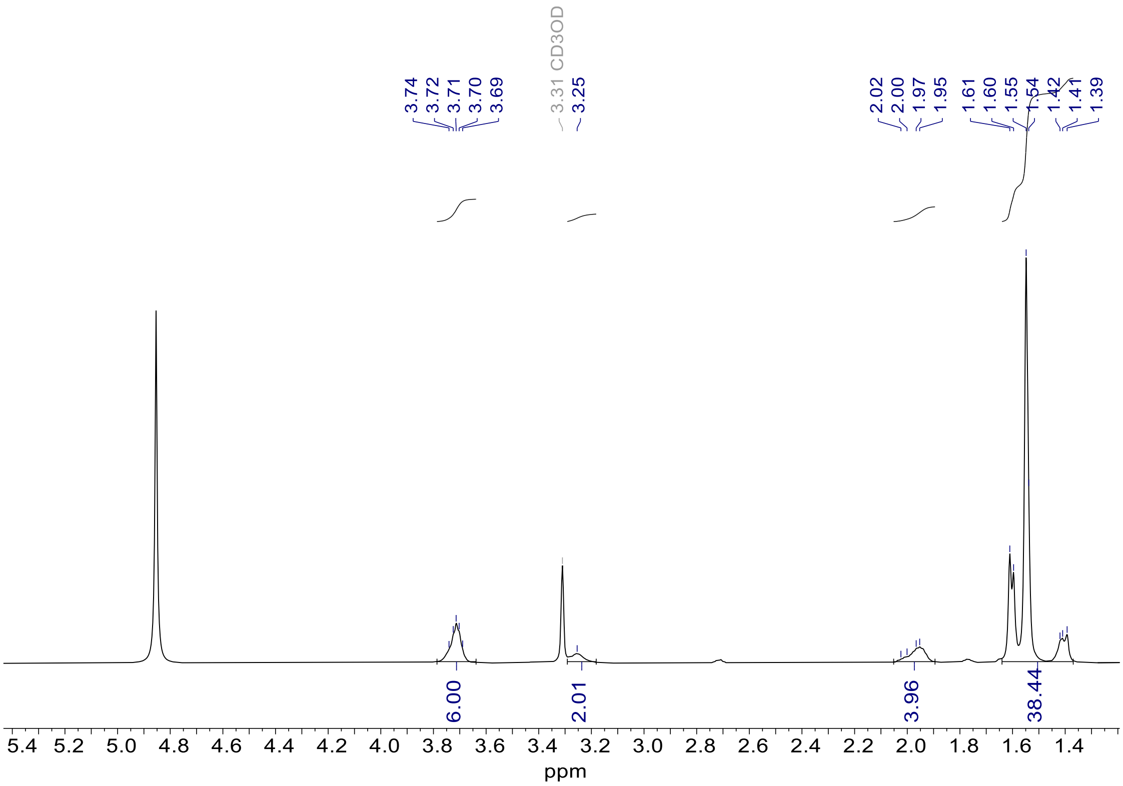

Figure S3. *^{1}H-NMR spectrum of complex **1b** in CD_3OD.*

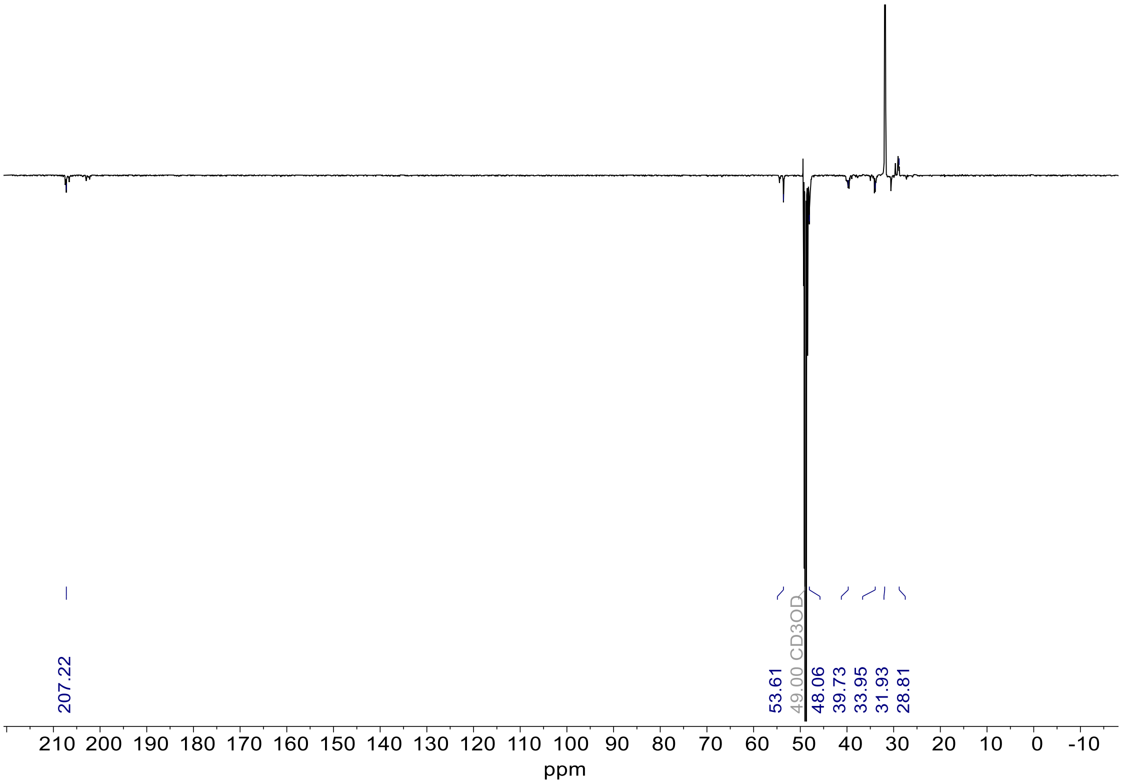

Figure S4. *^{13}C-NMR spectrum of complex **1b** in CD_3OD.*

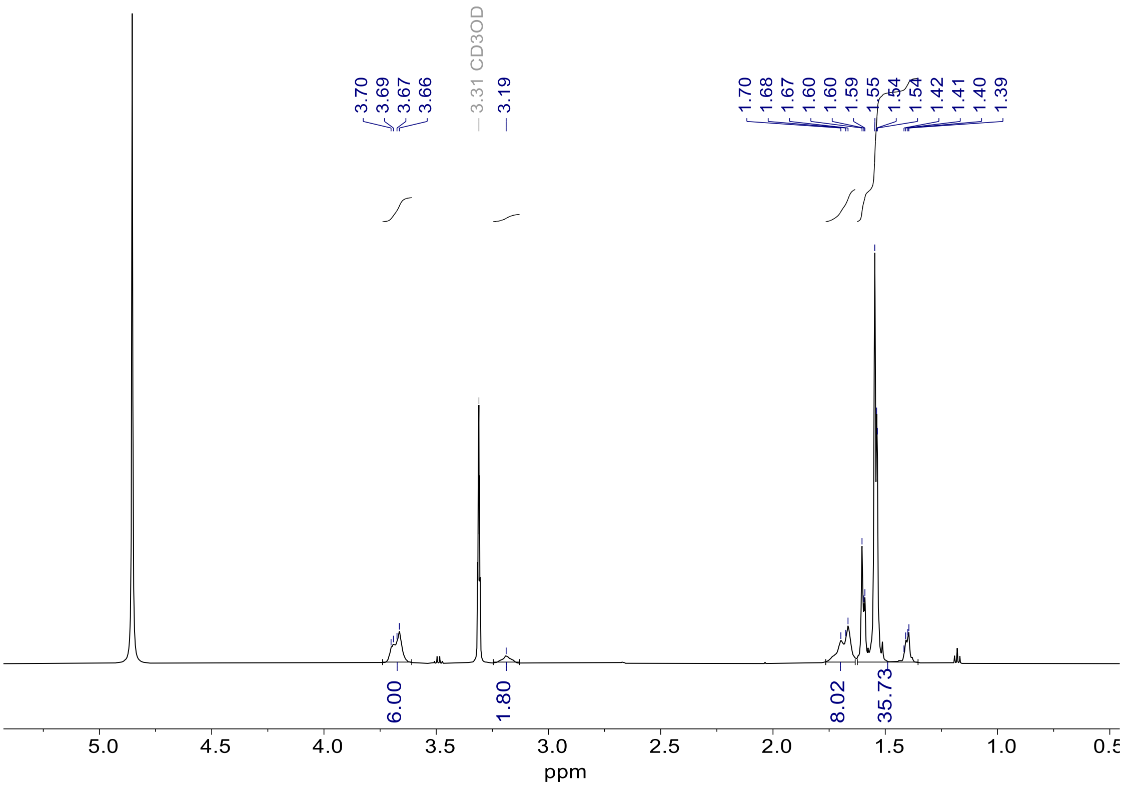

Figure S5. *^{1}H-NMR spectrum of complex **1c** in CD_3OD.*

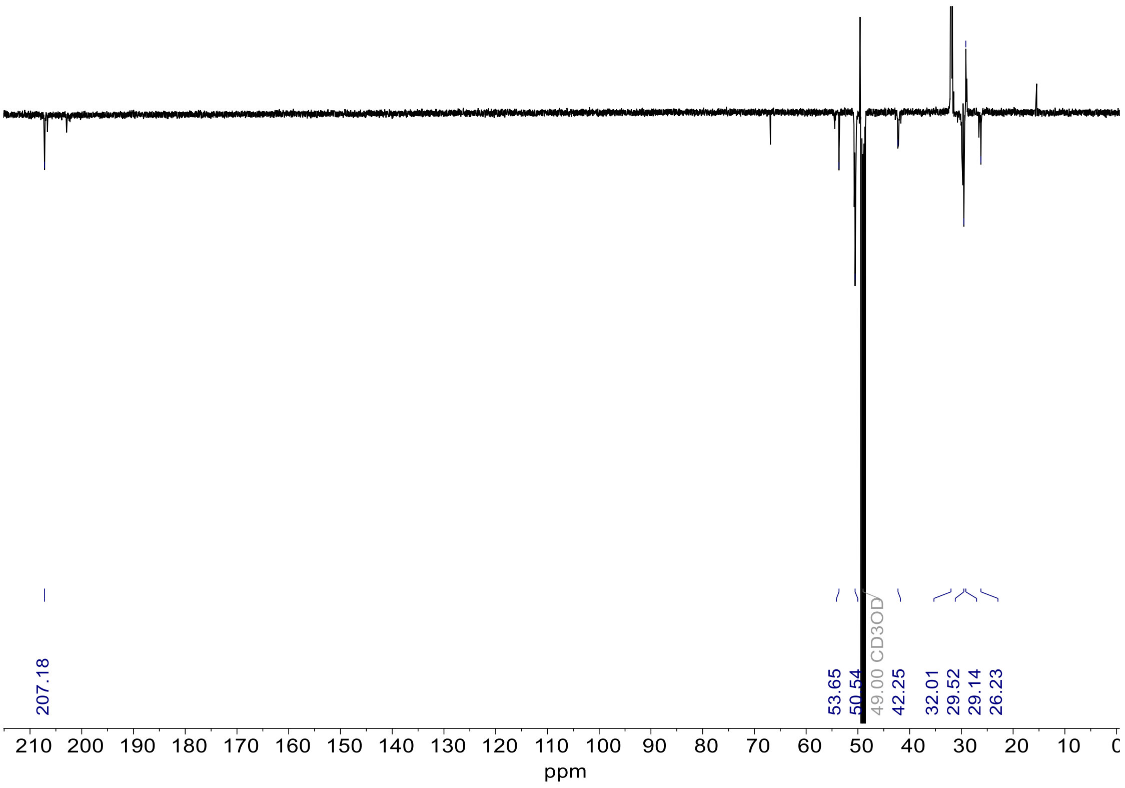

Figure S6. *^{13}C-NMR spectrum of complex **1c** in CD_3OD.*

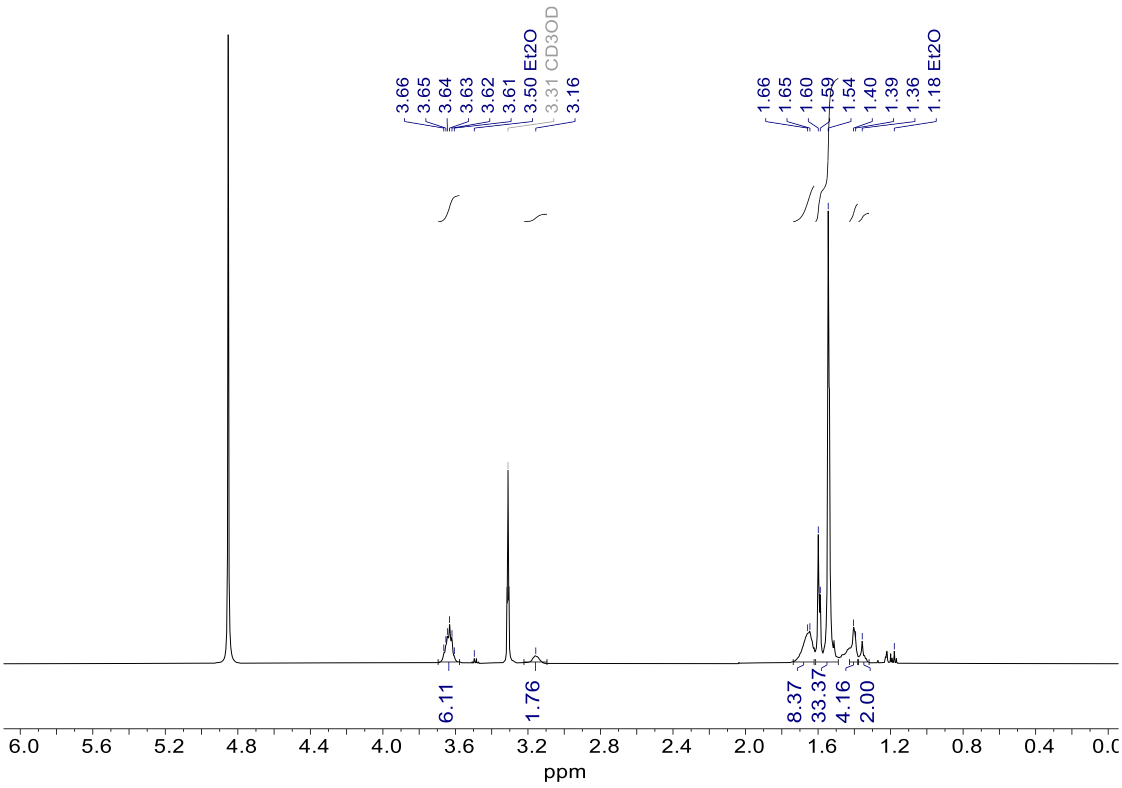

Figure S7. *^{1}H-NMR spectrum of complex **1d** in CD_3OD.*

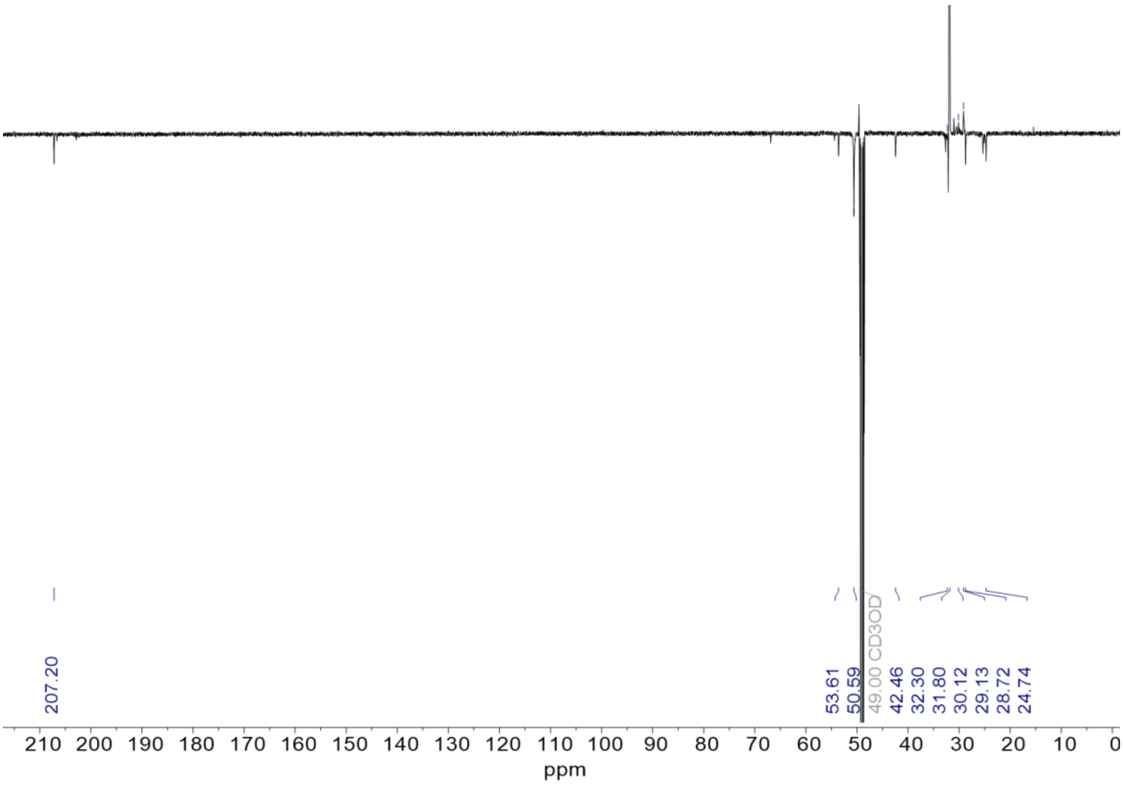

Figure S8. *^{13}C-NMR spectrum of complex **1d** in CD_3OD.*

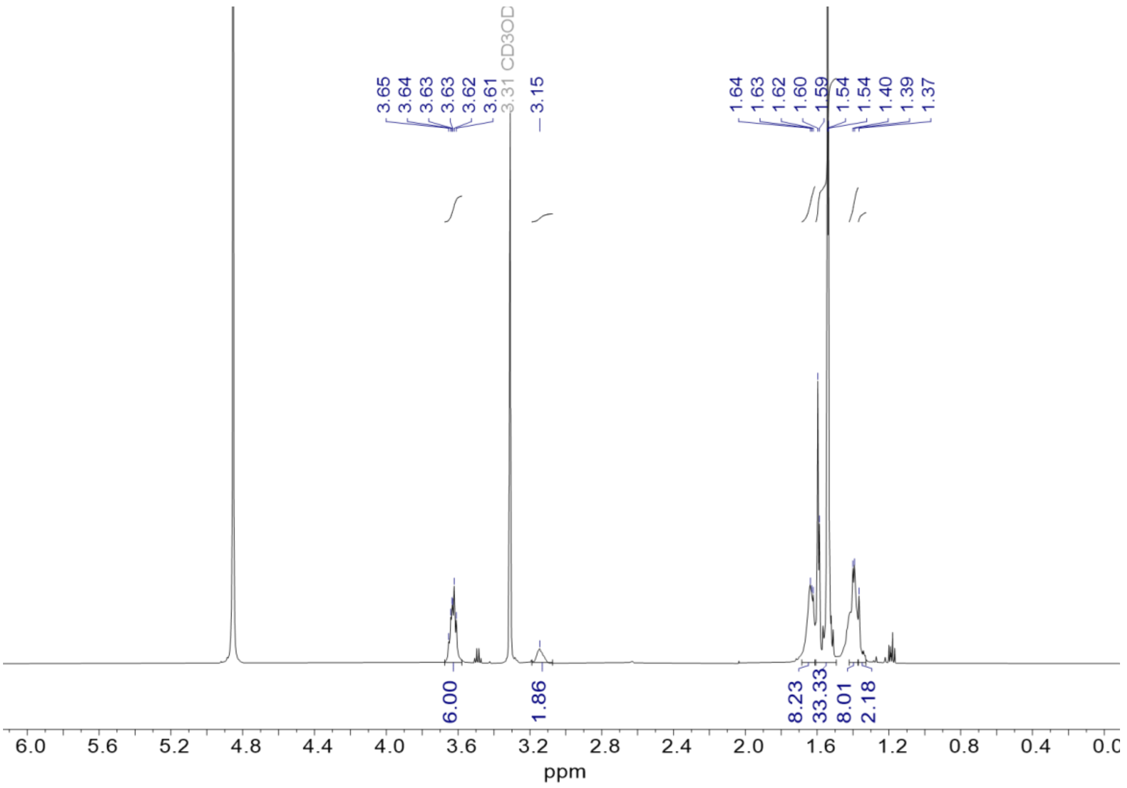

Figure S9. *^{1}H-NMR spectrum of complex **1e** in CD_3OD.*

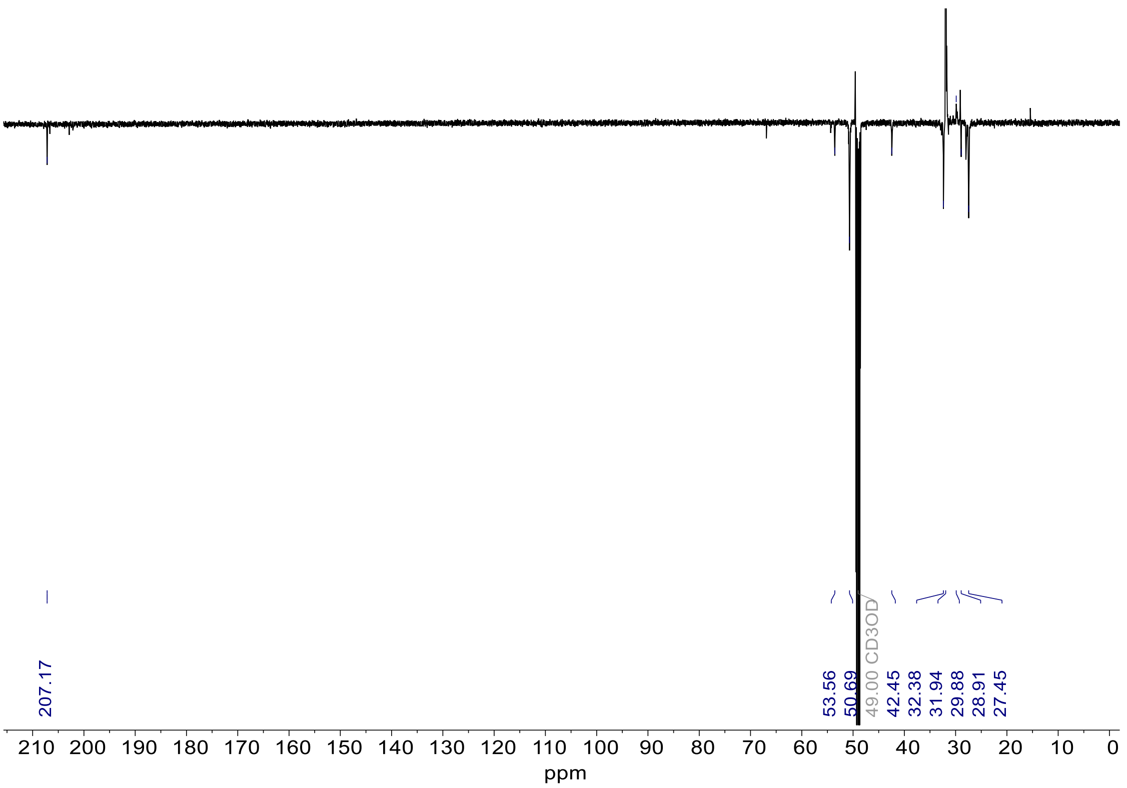

Figure S10. *^{13}C-NMR spectrum of complex **1e** in CD_3OD.*

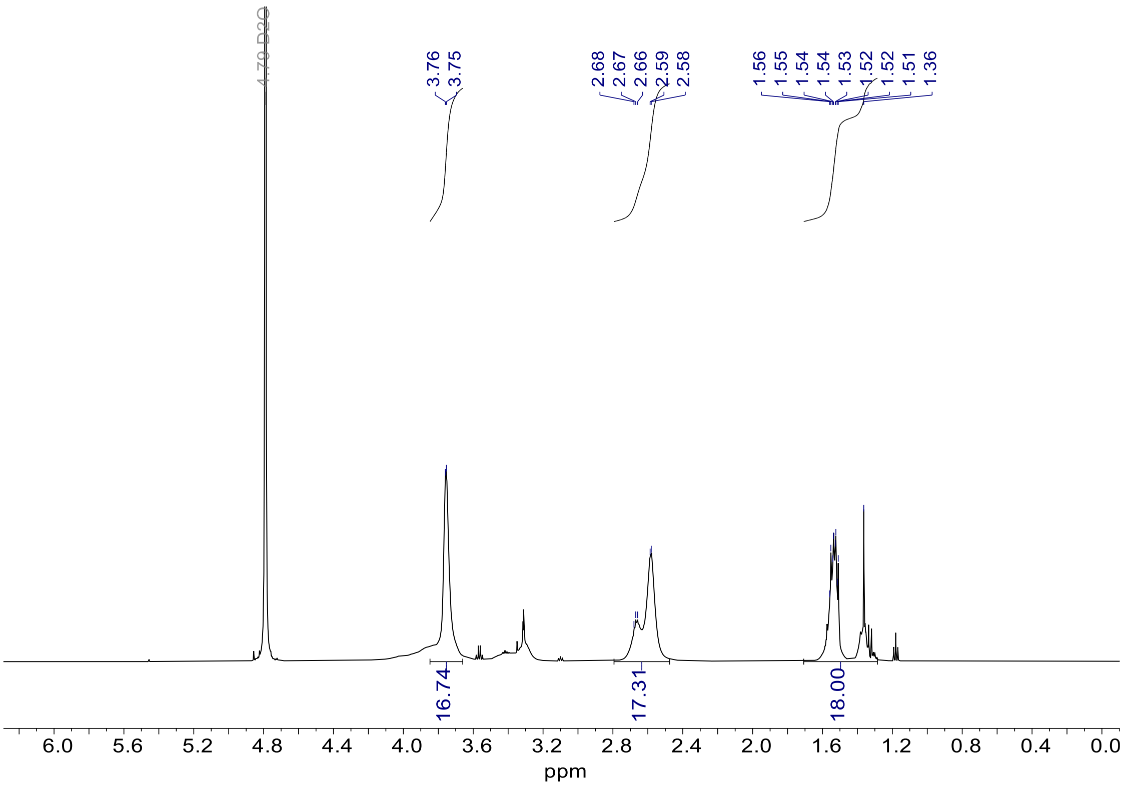

Figure S11. *^{1}H-NMR spectrum of complex **2a** in D_2O/CD_3OD.*

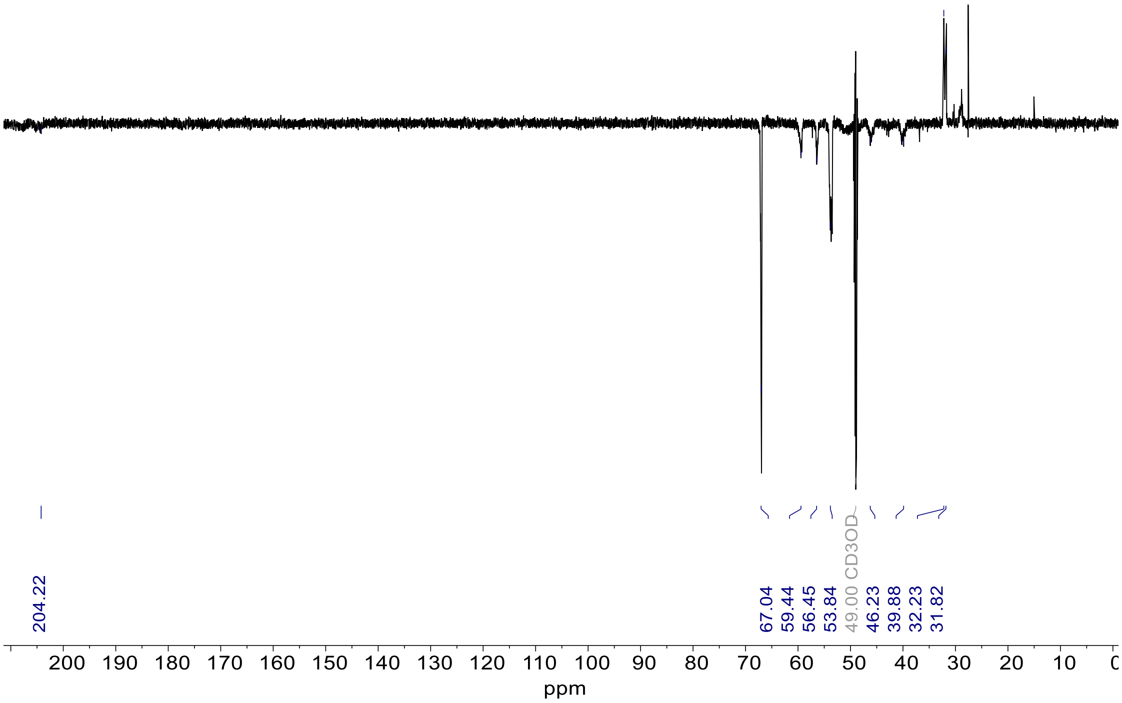

Figure S12. *^{13}C-NMR spectrum of complex **2a** in D_2O/CD_3OD.*

High-Resolution Electrospray Ionization Mass Spectrometry (HR-ESI-MS) of gold complexes

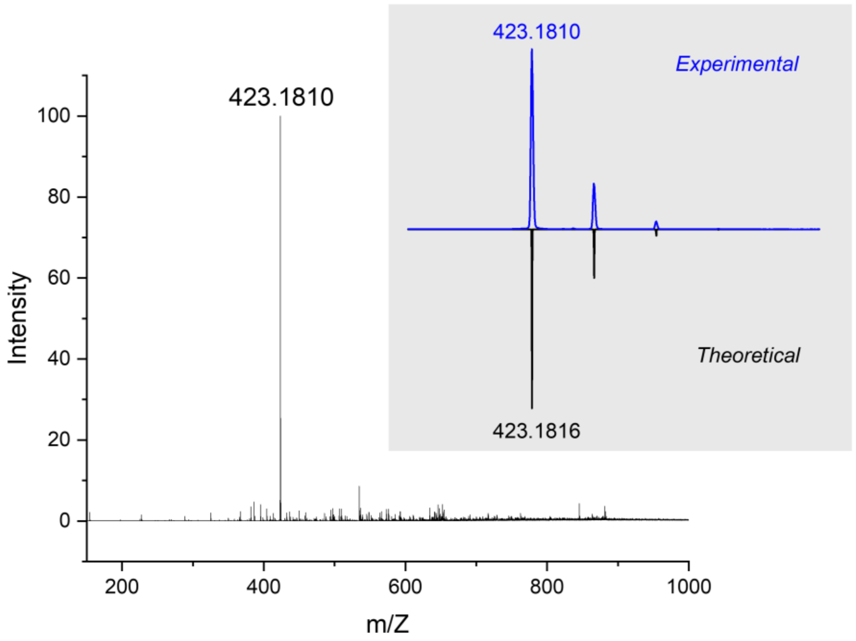

Figure S13. *HR-ESI-MS of complex **1a**: the inset shows the isotopic pattern of the species [M^{2+}] (m/Z 423.1810, mass error = -1.4178 ppm).*

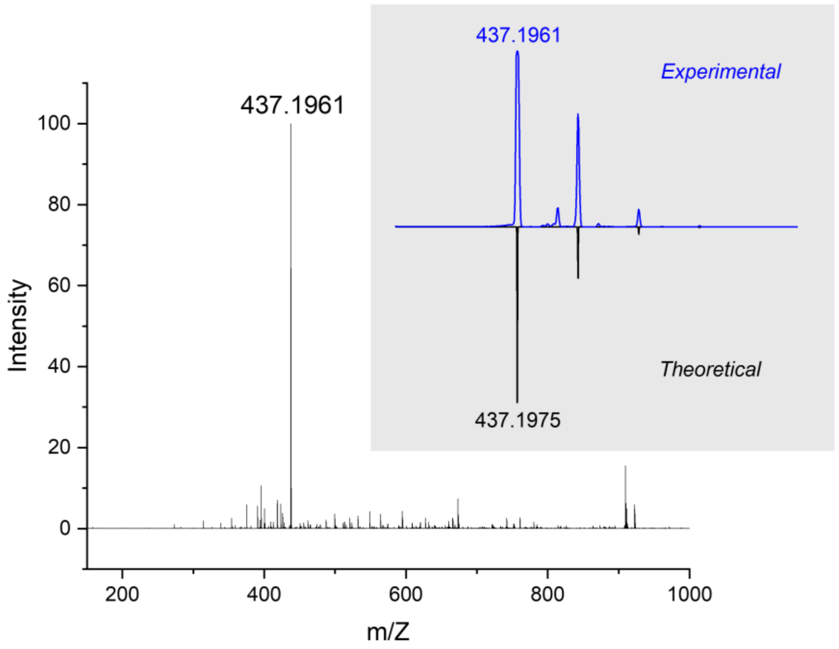

Figure S14. *HR-ESI-MS of complex **1b**: the inset shows the isotopic pattern of the species [M^{2+}] (m/Z 437.1961, mass error = -3.2022 ppm).*

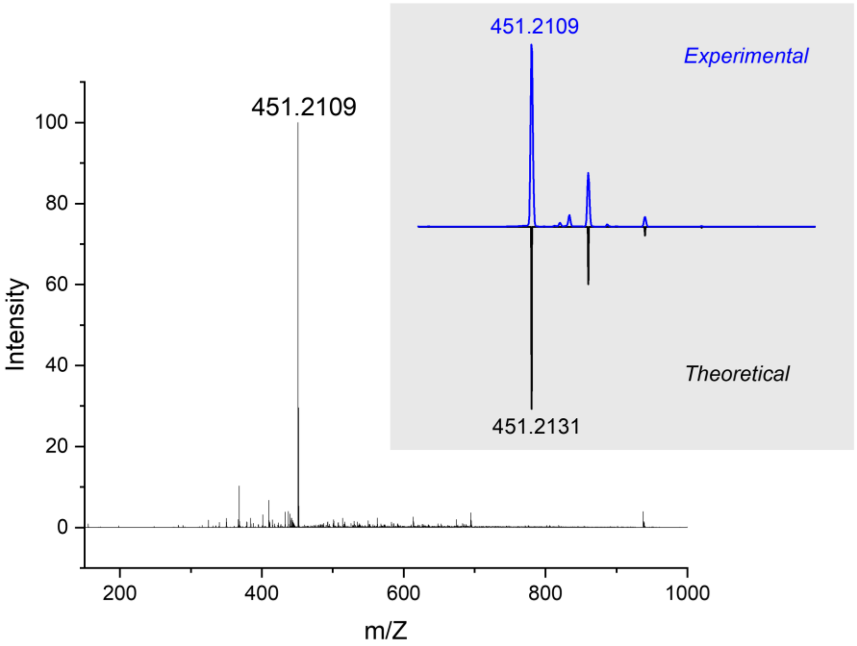

Figure S15. *HR-ESI-MS of complex* ***1c****: the inset shows the isotopic pattern of the species [M^{2+}] (m/Z 451.2109, mass error = -4.8757 ppm).*

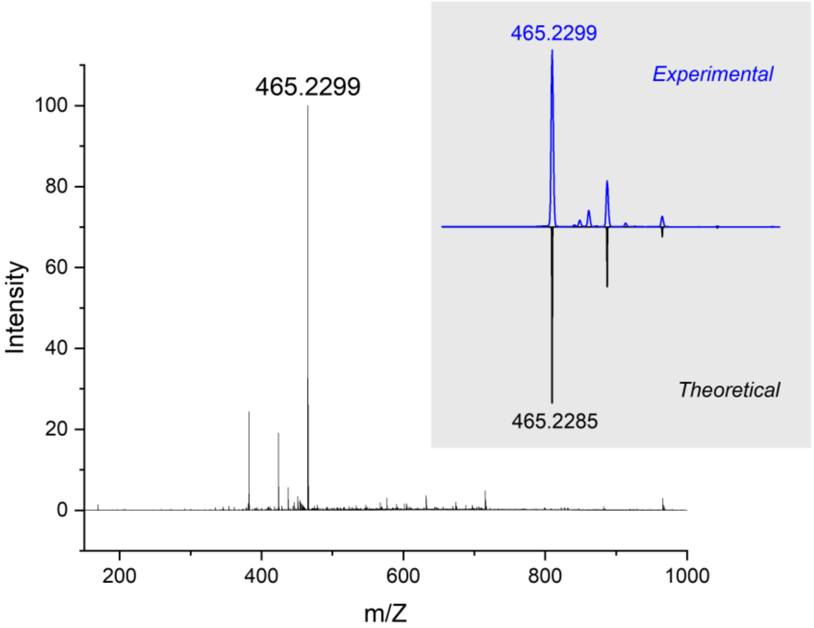

Figure S16. *HR-ESI-MS of complex* ***1d****: the inset shows the isotopic pattern of the species [M^{2+}] (m/Z 465.2299, mass error = -4.8757 ppm).*

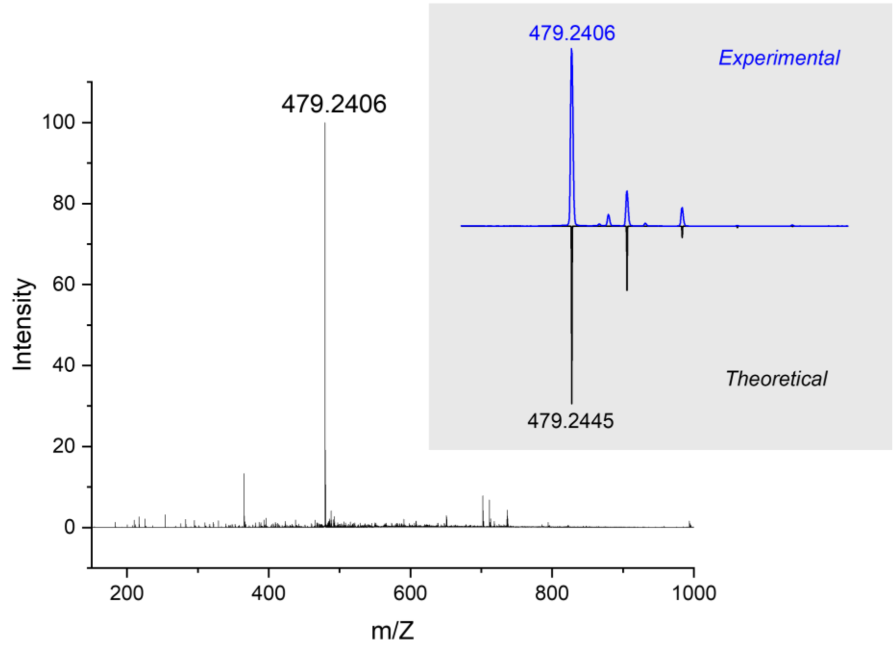

Figure S17. *HR-ESI-MS of complex* ***1e****: the inset shows the isotopic pattern of the species [M^{2+}] (m/Z 479.2406, mass error = -8.1378 ppm).*

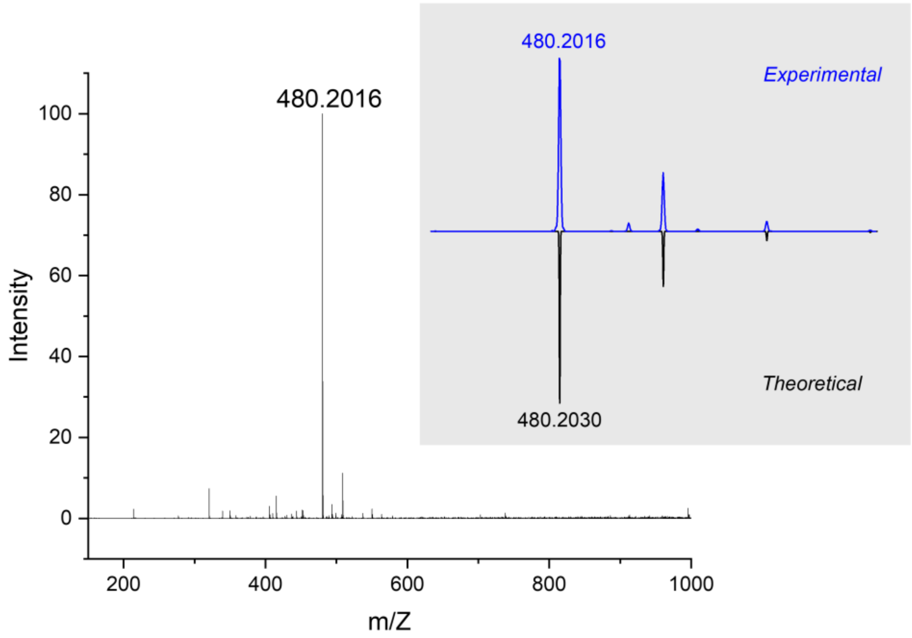

Figure S18. *HR-ESI-MS of complex* ***2a****: the inset shows the isotopic pattern of the species [M^{2+}] (m/Z 480.2016, mass error = -2.9154 ppm).*

Crystallographic Data

Complex **1a** was crystallized by slow diffusion of ethyl acetate from a pyridine solution containing **1a** dissolved. The asymmetric unit contains half a molecule of **1a**, with the second half being generated about an inversion center. The asymmetric unit also contains half a molecule of pyridine and half a molecule of ethyl acetate, which are substitutionally disordered at the same position. CCDC no: 2425841.

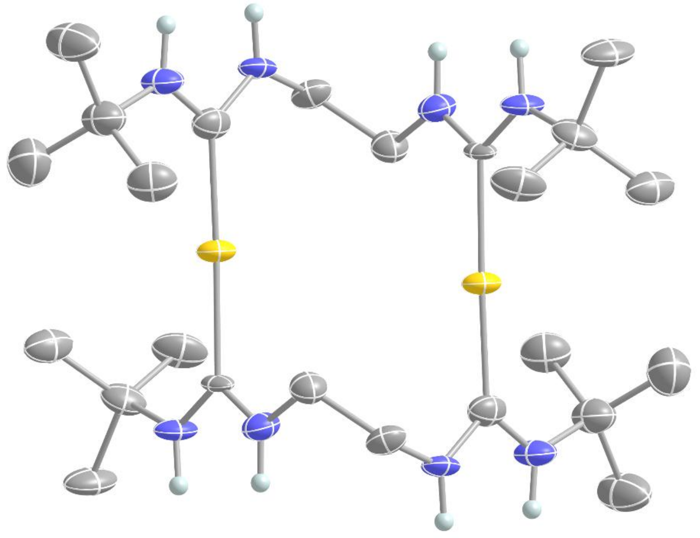

Figure S19. *Crystallographic structure of compound **1a**.*

Table 1. Crystallographic data for [1a·C_5H_5N·EtOAc].

Empirical formula	$C_{33}H_{65}Au_2Cl_2N_9O_2$
Formula weight	1084.77
Temperature/K	100(2)
Crystal system	orthorhombic
Space group	*Pbca*
a/Å	18.4016(8)
b/Å	11.5547(5)
c/Å	20.2224(9)
α/°	90
β/°	90
γ/°	90
V/Å^3	4299.8(3)
Z	4
ρ_{calc}g/cm^3	1.676
μ/mm^{-1}	6.979
F(000)	2136.0
Crystal size/mm^3	0.18 × 0.1 × 0.05
Wavelength	0.71073 Å
2 theta range for data collection/°	4.428 to 60.106
Index ranges	-23 ≤ h ≤ 25, -15 ≤ k ≤ 16, -28 ≤ l ≤ 27
Reflections collected	33698
Independent reflections	6298 [R_{int} = 0.0825, R_{sigma} = 0.0647]
Data/restraints/parameters	6298/238/256
Goodness-of-fit on F^2	1.088
Final R indexes [I≥2σ (I)]	R_1 = 0.0654, wR_2 = 0.1639
Final R indexes [all data]	R_1 = 0.1082, wR_2 = 0.1810
Largest diff. peak/hole / e Å^{-3}	5.56/-3.11

Complex **1d** was crystallized by slow evaporation from methanol. The asymmetric unit contains half a molecule of **1d**, with the second half being generated about a two-fold rotation axis. The asymmetric unit also contains half a molecule of methanol which is disordered about an inversion center. CCDC no: 2425840.

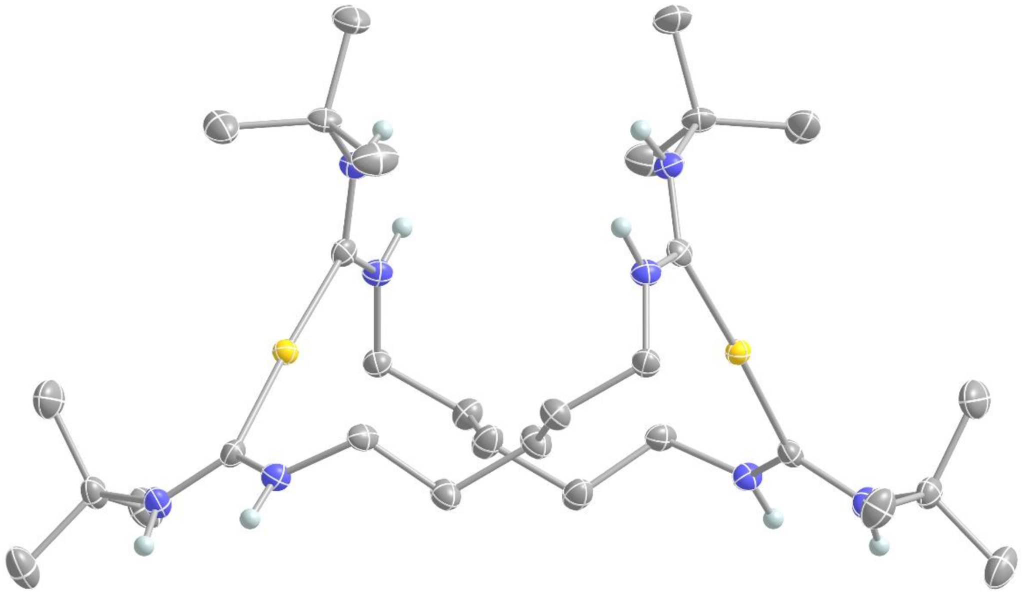

Figure S20. *Crystallographic structure of compound **1d**.*

Table 2. Crystallographic data for [1d·MeOH].

Empirical formula	$C_{31}H_{68}N_8OCl_2Au_2$
Formula weight	1033.76
Temperature/K	100(2)
Crystal system	monoclinic
Space group	*P*2/*c*
a/Å	14.1431(9)
b/Å	9.4921(4)
c/Å	16.4833(9)
α/°	90
β/°	113.494(5)
γ/°	90
V/Å^3	2029.4(2)
Z	2
ρ_{calc}g/cm^3	1.692
μ/mm^{-1}	7.386
F(000)	1020.0
Crystal size/mm^3	0.14 × 0.06 × 0.06
Wavelength	0.71073 Å
2 theta range for data collection/°	5.042 to 62.572
Index ranges	-19 ≤ h ≤ 20, -13 ≤ k ≤ 13, -23 ≤ l ≤ 21
Reflections collected	32291
Independent reflections	6356 [R_{int} = 0.0435, R_{sigma} = 0.0463]
Data/restraints/parameters	6356/4/210
Goodness-of-fit on F^2	0.893
Final *R* indexes [$I \geq 2\sigma(I)$]	R_1 = 0.0273, wR_2 = 0.0568
Final *R* indexes [all data]	R_1 = 0.0481, wR_2 = 0.0631
Largest diff. peak/hole / e Å^{-3}	1.36/-2.29

UV-Vis of ADC-AuNPs

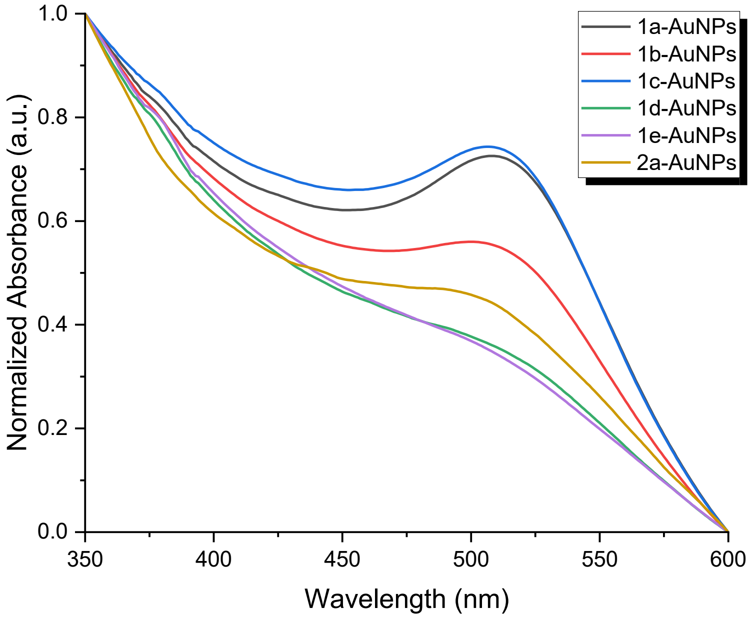

Figure S21. *UV-Vis of the series **1a-AuNPs** to **1e-AuNPs** in methanol and **2a-AuNPs** in MilliQ* H_2O.

^{1}H and ^{13}C NMR Spectroscopy of ADC-AuNPs

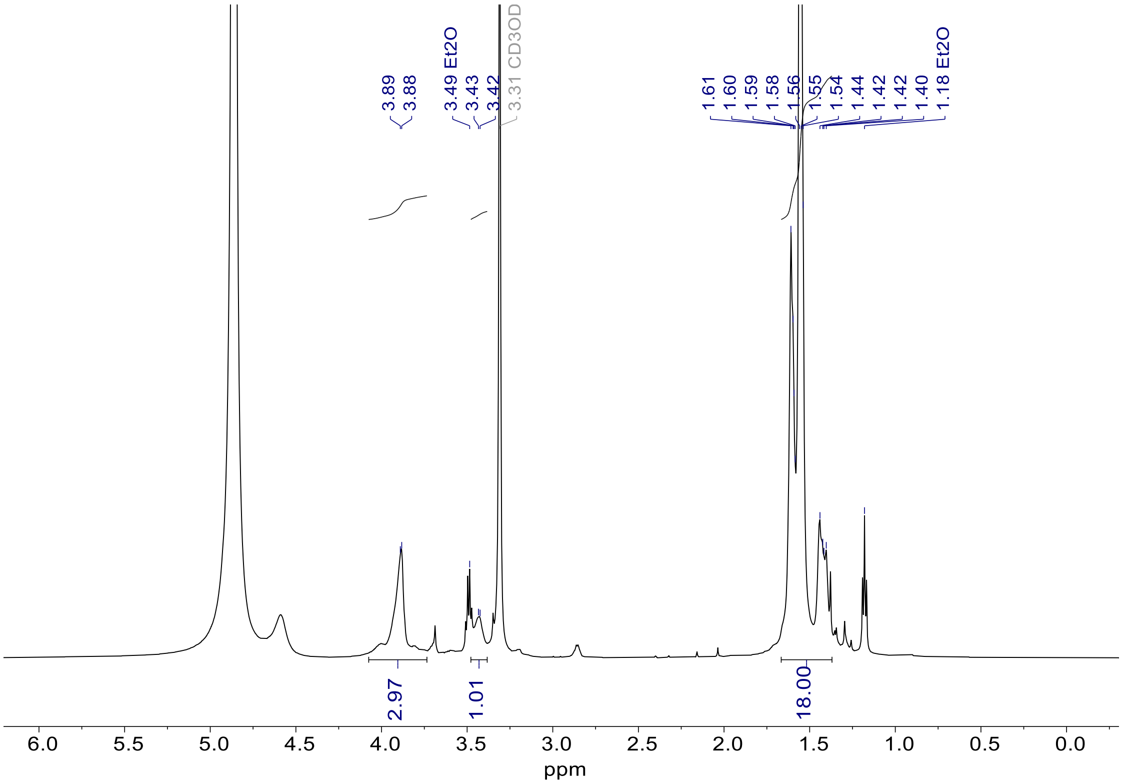

Figure S22. *^{1}H-NMR spectrum of* ***1a-AuNPs*** *in CD_3OD.*

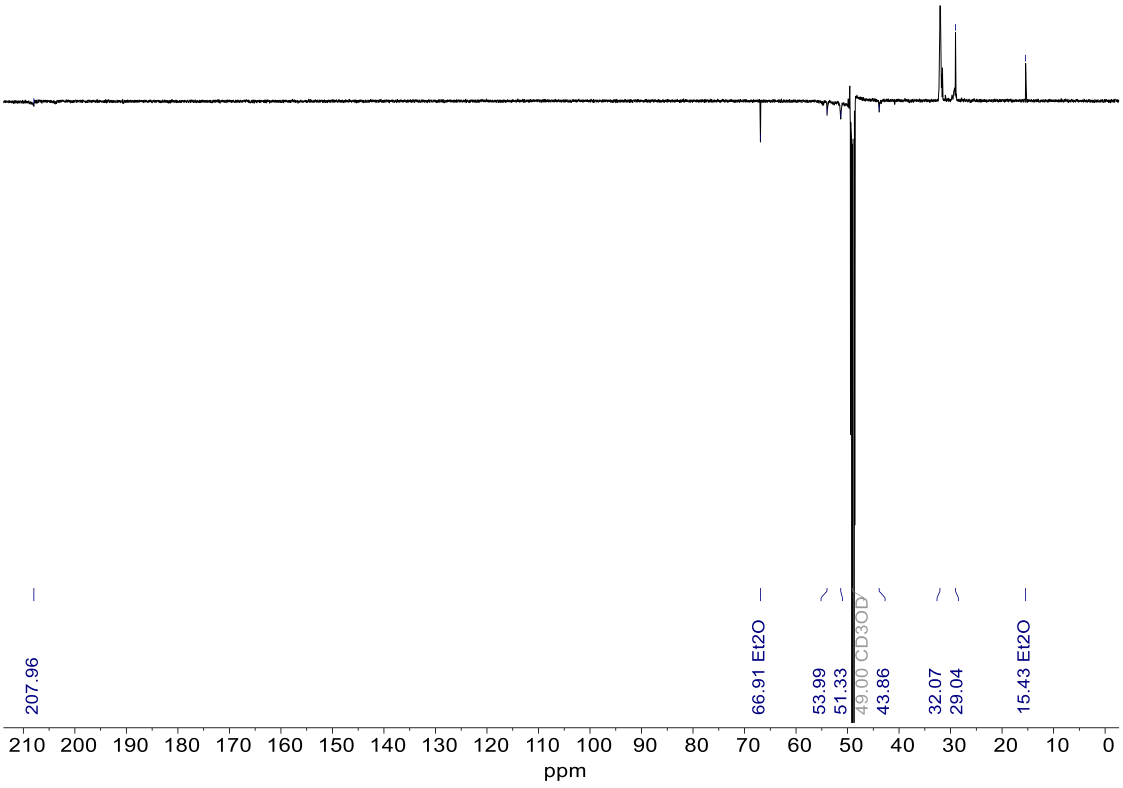

Figure S23. *^{13}C-NMR spectrum of* ***1a-AuNPs*** *in CD_3OD.*

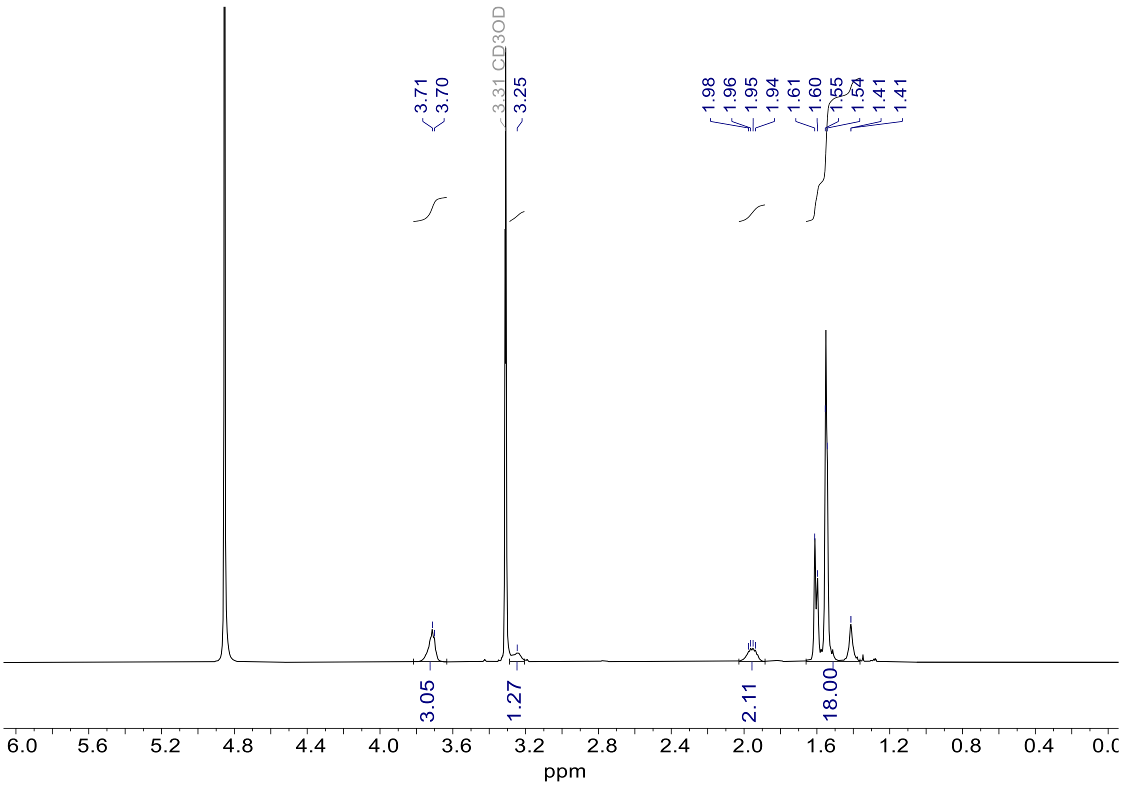

Figure S24. *^{1}H-NMR spectrum of* ***1b-AuNPs*** *in CD_3OD.*

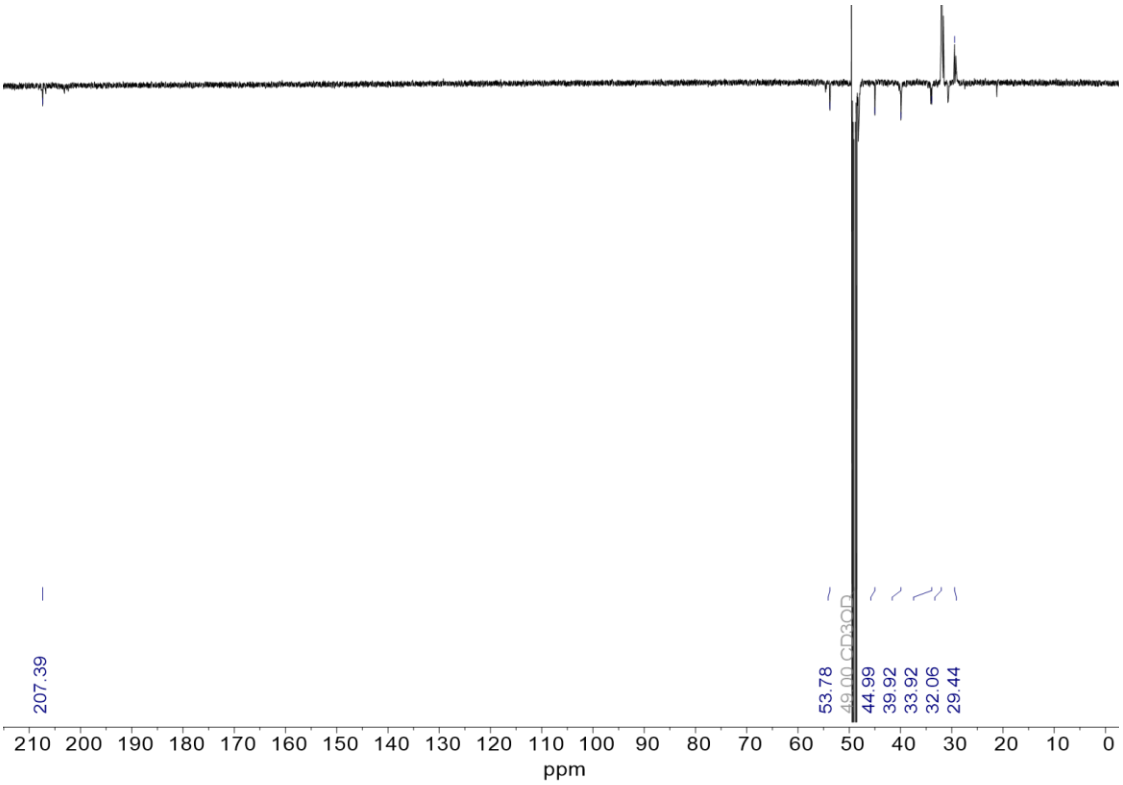

Figure S25. *^{13}C-NMR spectrum of* ***1b-AuNPs*** *in CD_3OD.*

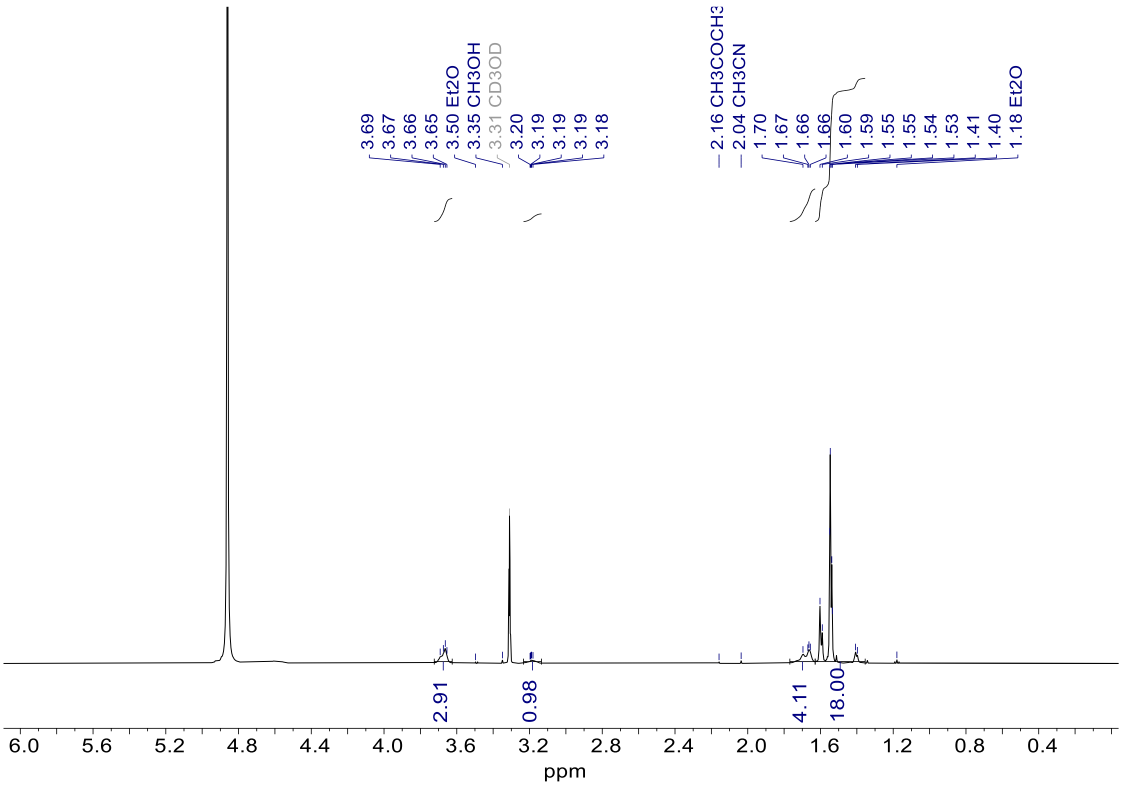

Figure S26. *^{1}H-NMR spectrum of **1c-AuNPs** in CD_3OD.*

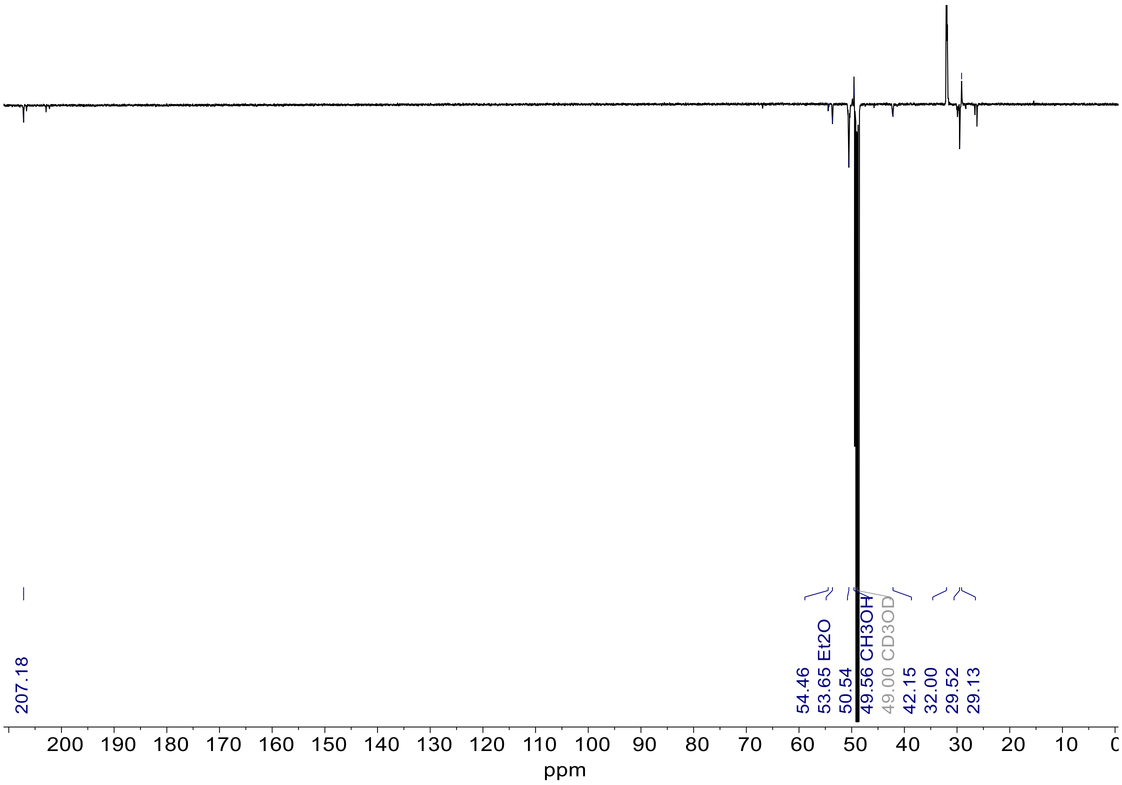

Figure S27. *^{13}C-NMR spectrum of **1c-AuNPs** in CD_3OD.*

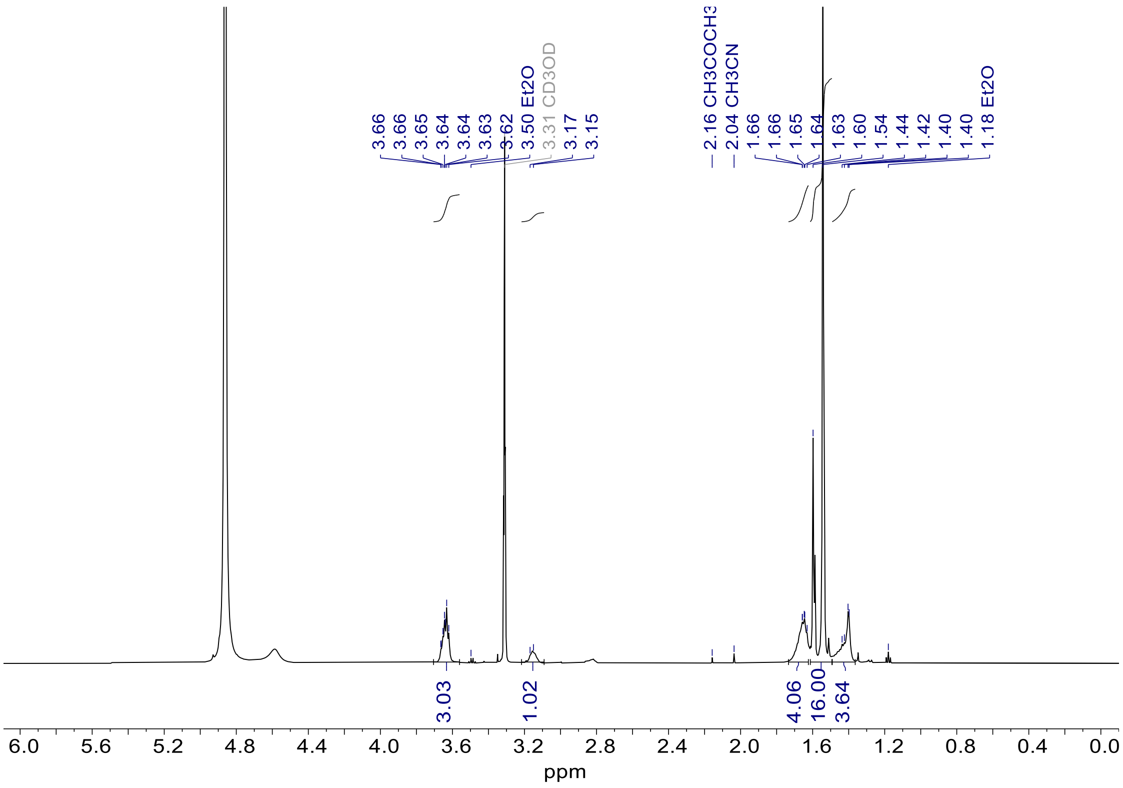

Figure S28. *^{1}H-NMR spectrum of* ***1d-AuNPs*** *in CD_3OD.*

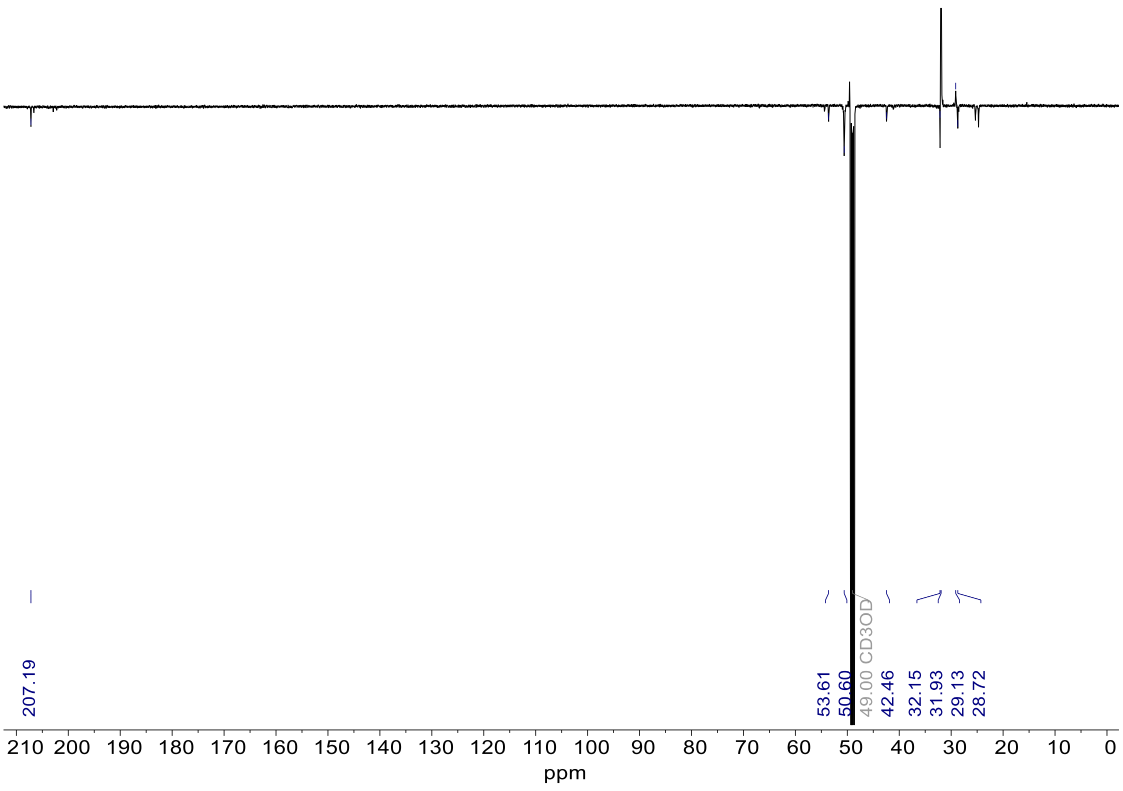

Figure S29. *^{13}C-NMR spectrum of* ***1d-AuNPs*** *in CD_3OD.*

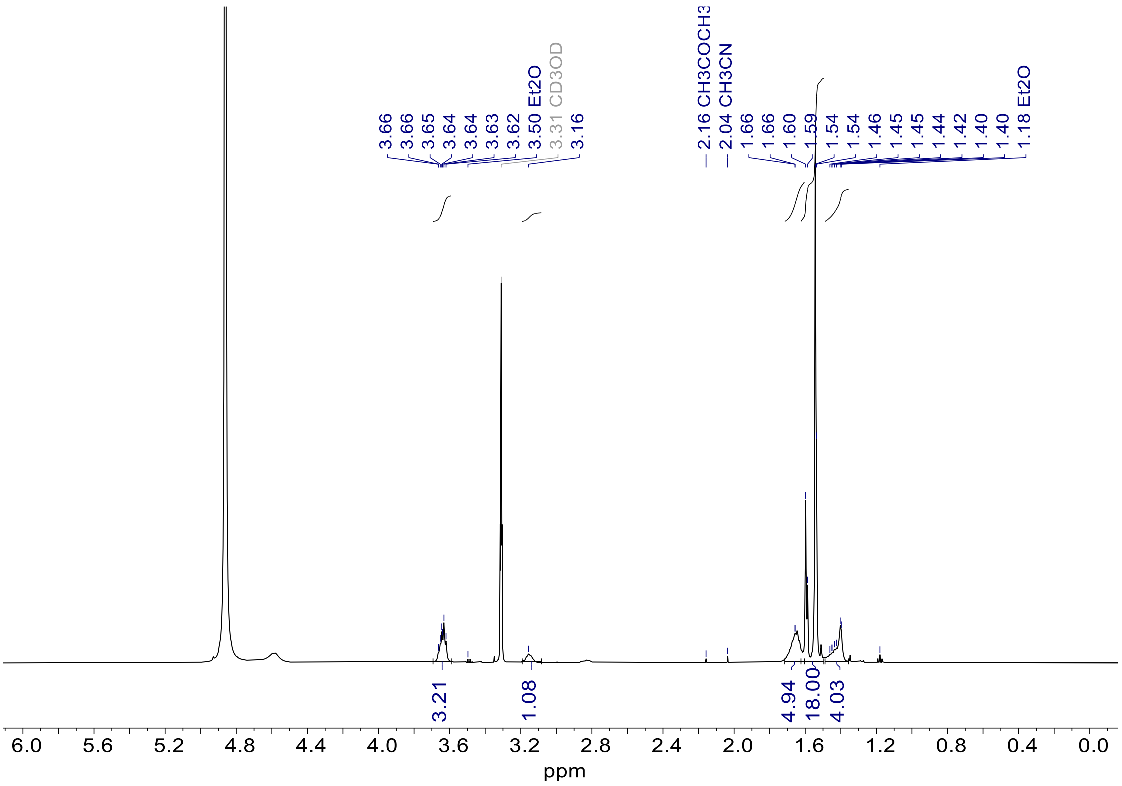

Figure S30. *^{1}H-NMR spectrum of **1e-AuNPs** in CD_3OD.*

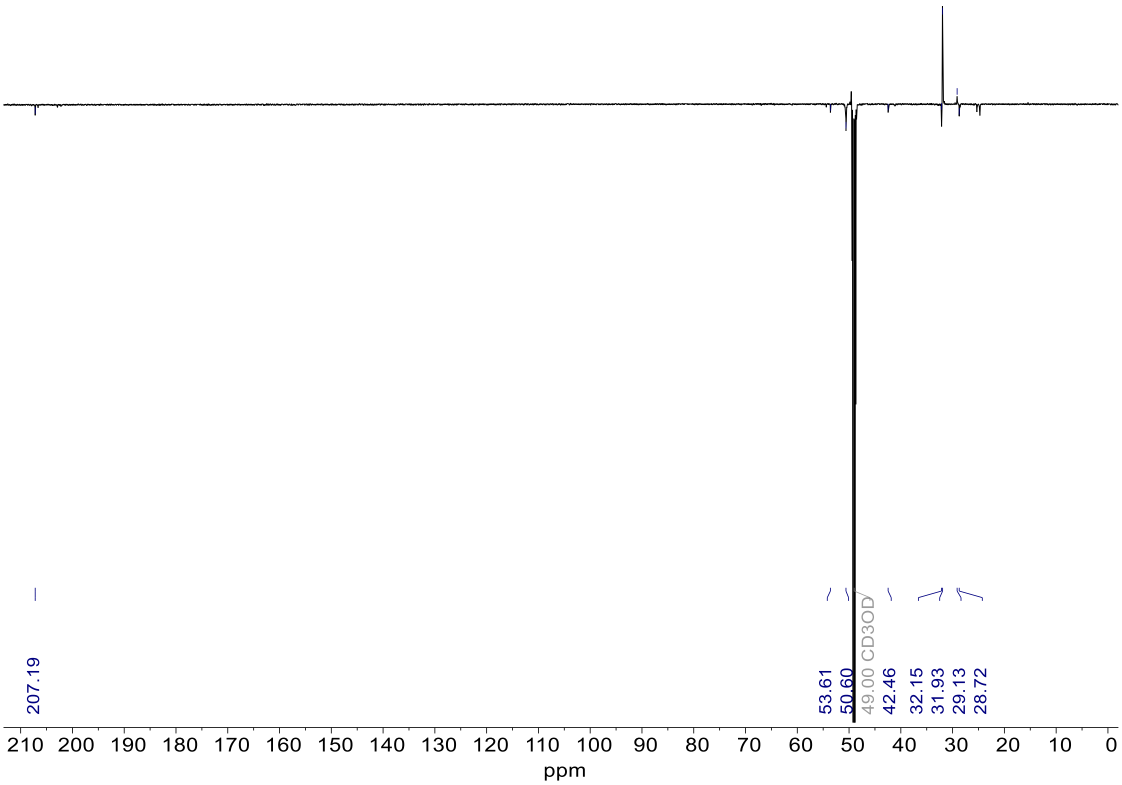

Figure S31. *^{13}C-NMR spectrum of **1e-AuNPs** in CD_3OD.*

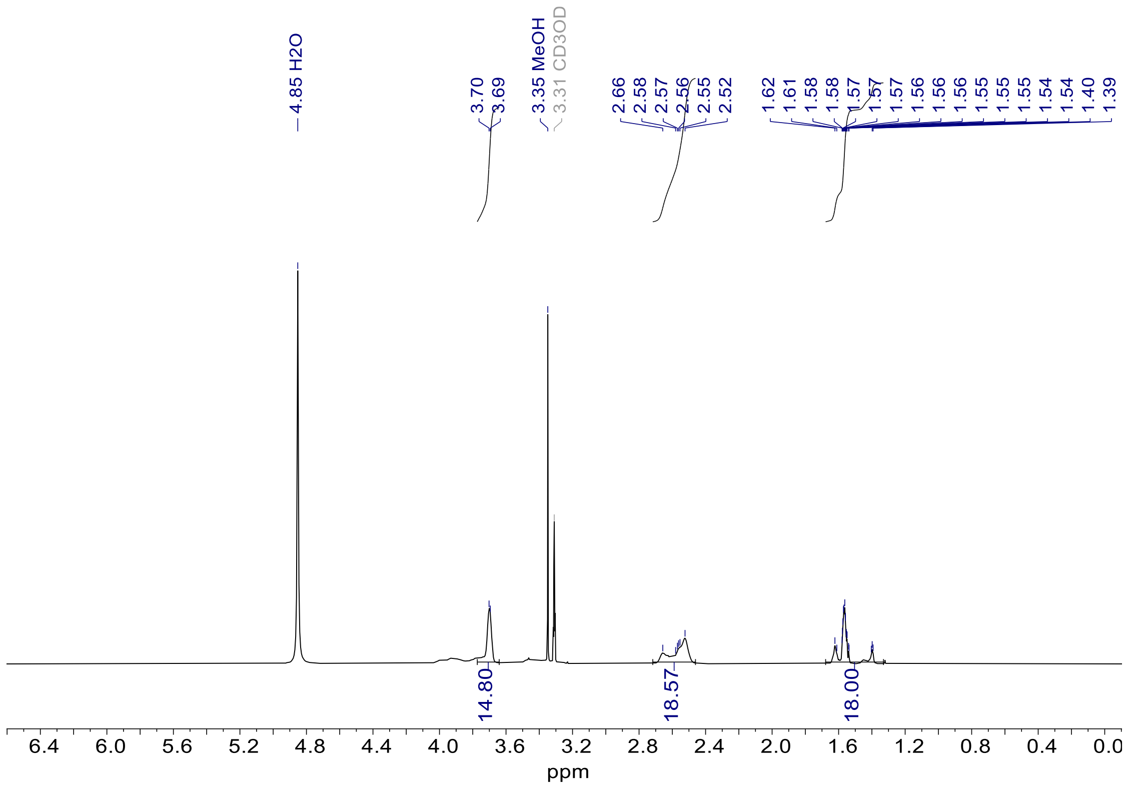

***Figure S32.** ^{1}H-NMR spectrum of **2a-AuNPs** in CD_3OD.*

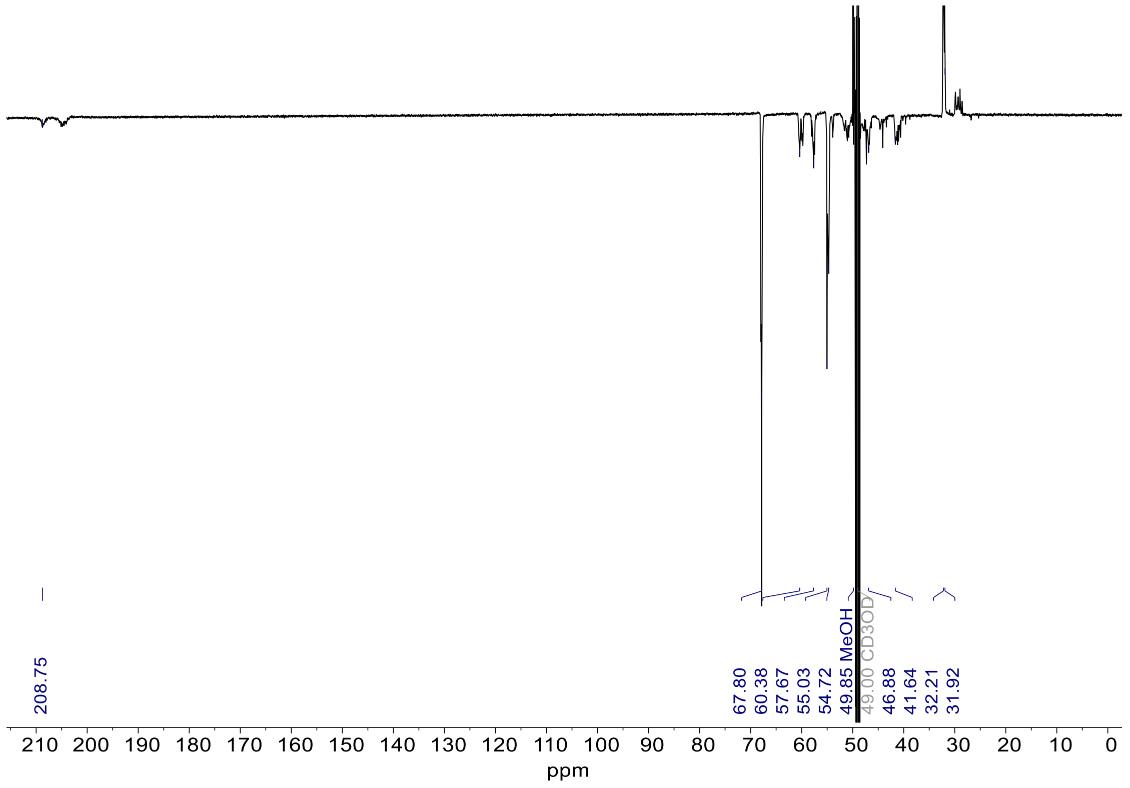

***Figure S33.** ^{13}C-NMR spectrum of **2a-AuNPs** in CD_3OD.*

Transmission Electron Microscopy (TEM) Images of ADC-AuNPs

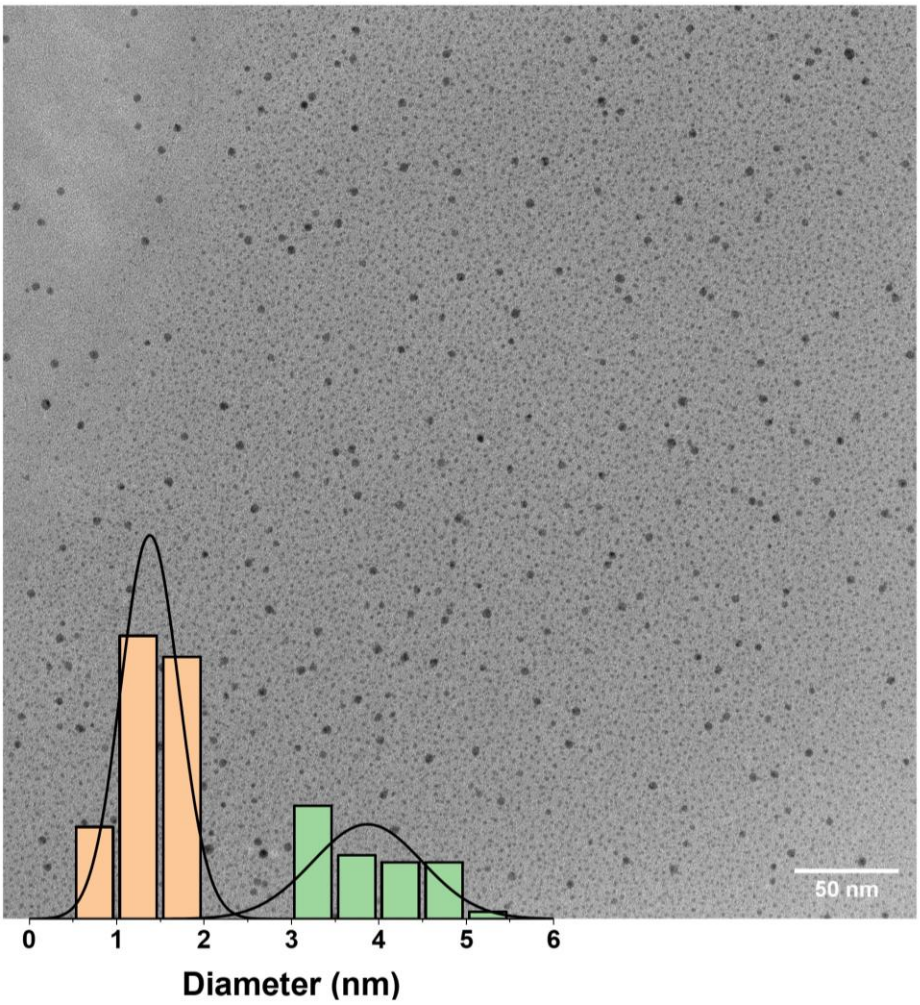

Figure S34. *TEM micrograph of **1a-AuNPs** with average size histogram. Overall average particle size measured = 3.16 ± 0.73 nm, which can be split into two different groups (1.38 ± 0.33 nm and 3.87 ± 0.63 nm).*

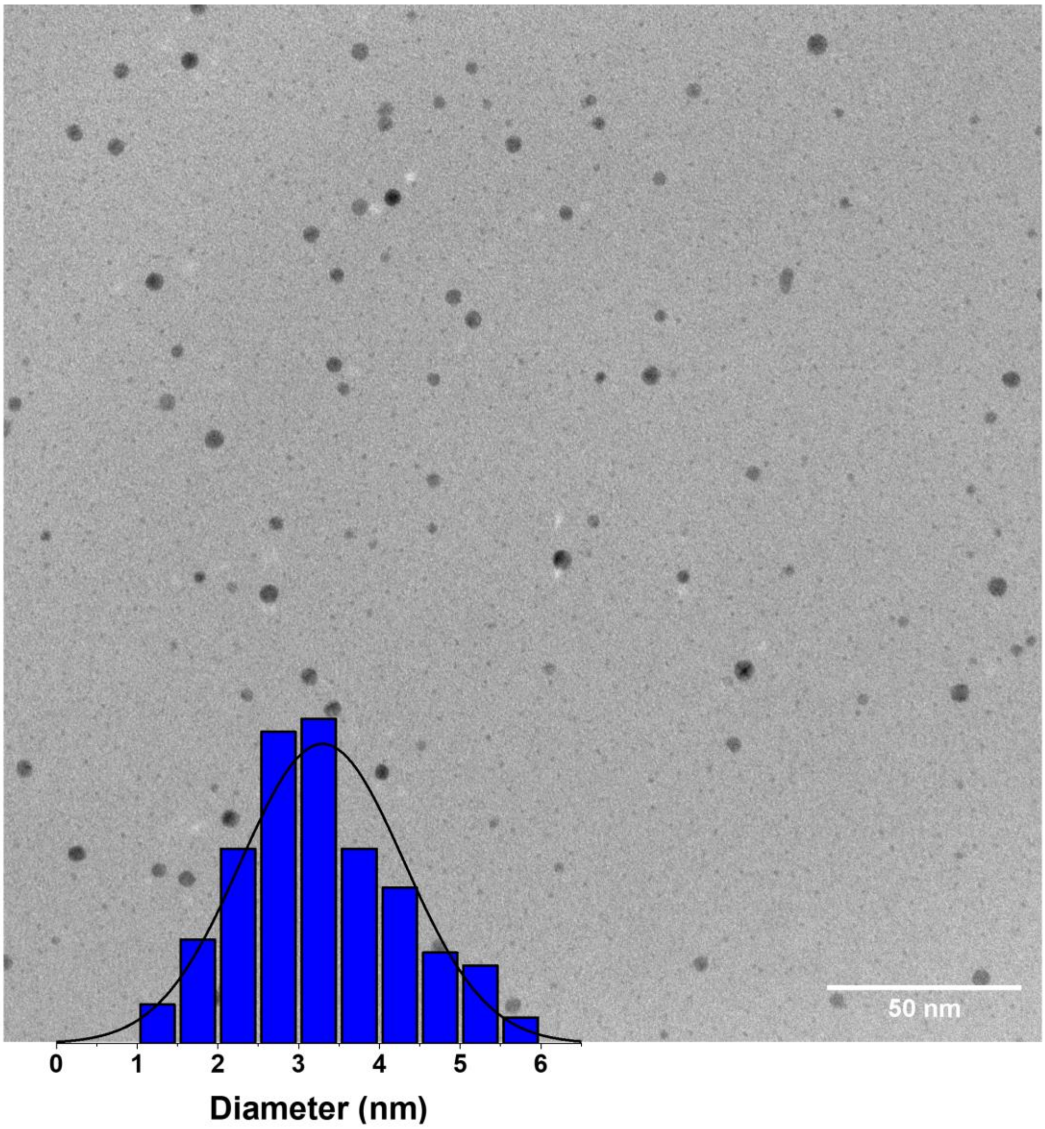

Figure S35. *TEM micrograph of* ***1b-AuNPs*** *with average size histogram. Average particle size measured = 3.29 ± 1.01 nm.*

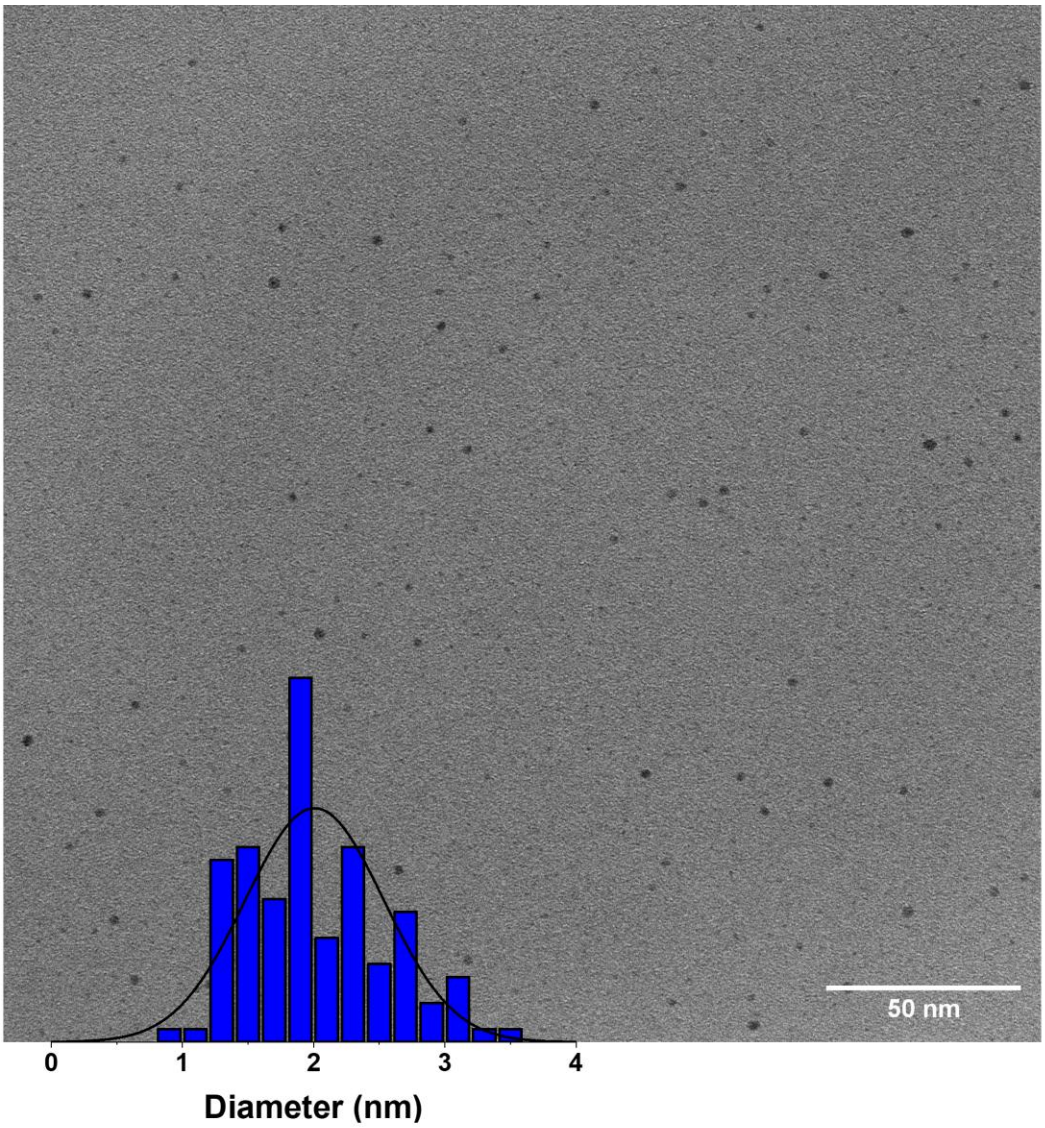

Figure S36. *TEM micrograph of **1c-AuNPs** with average size histogram. Average particle size measured = 2.01 ± 0.53 nm.*

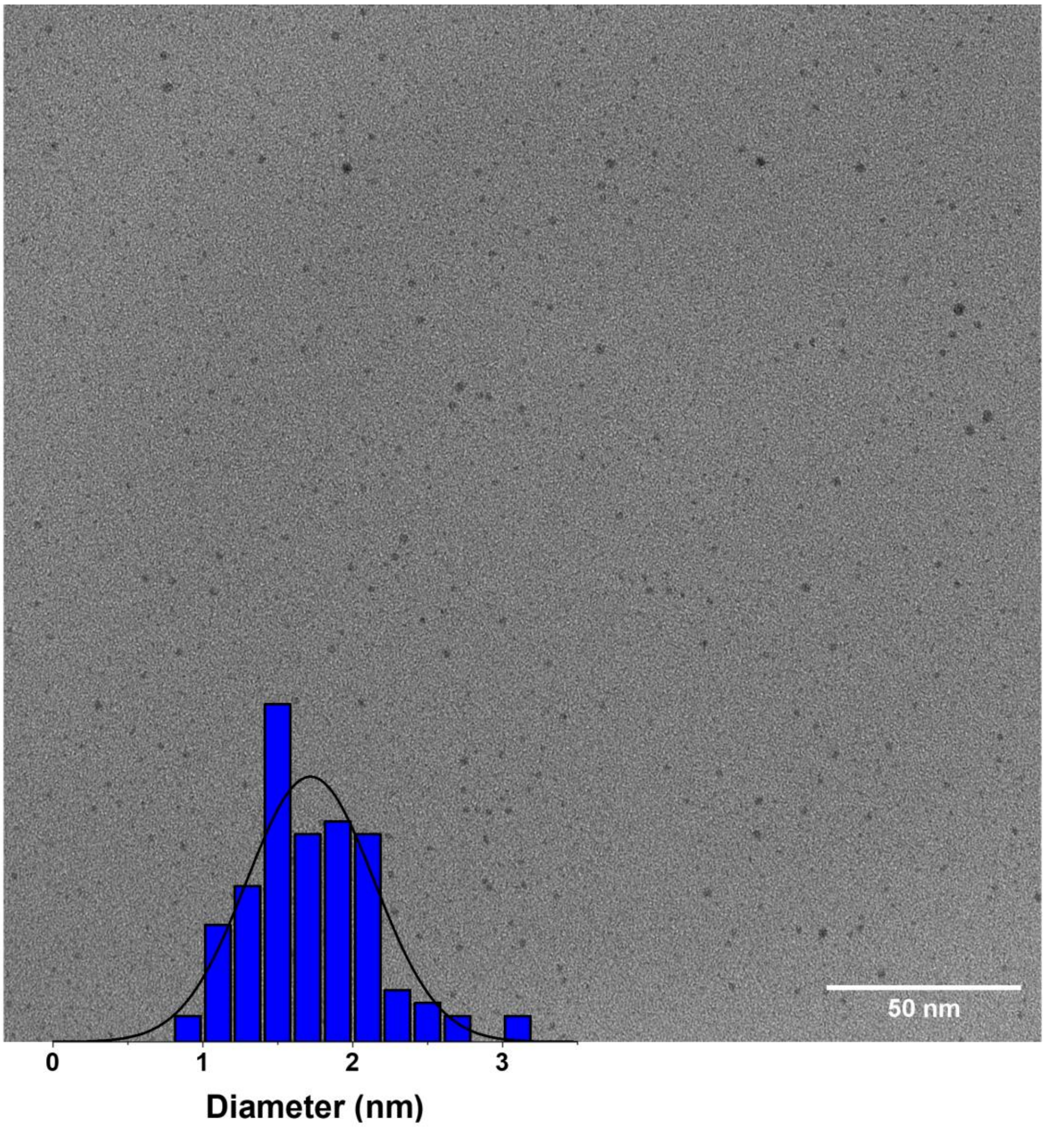

Figure S37. *TEM micrograph of **1d-AuNPs** with average size histogram. Average particle size measured = 1.72 ± 0.43 nm.*

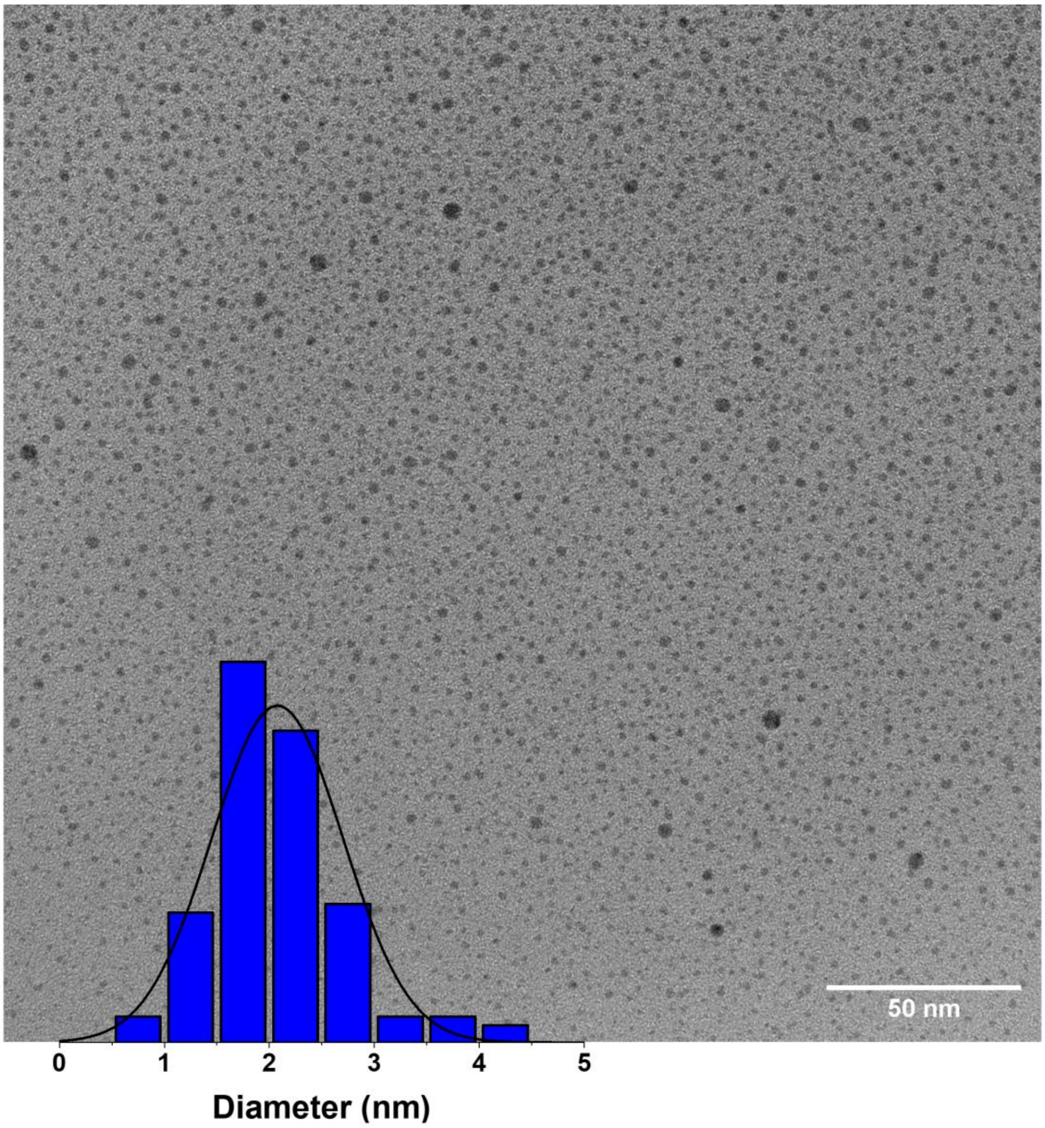

Figure S38. *TEM micrograph of **1e-AuNPs** with average size histogram. Average particle size measured = 2.08 ± 0.63 nm.*

Thermogravimetric Analysis (TGA) of ADC-AuNPs

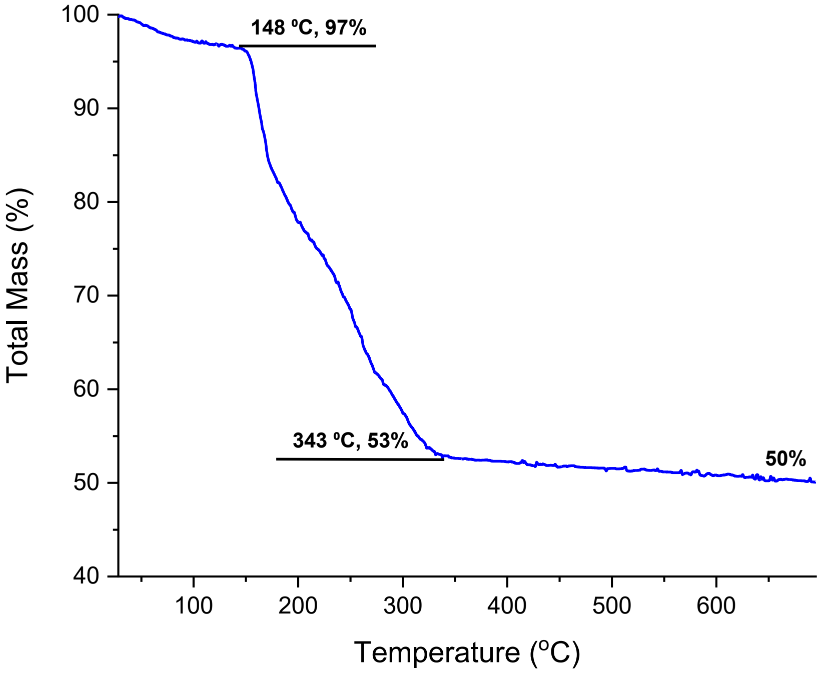

Figure S39. *Thermogravimetric analysis of **1a-AuNPs**. Overall organic ligand content is estimated to be 47%.*

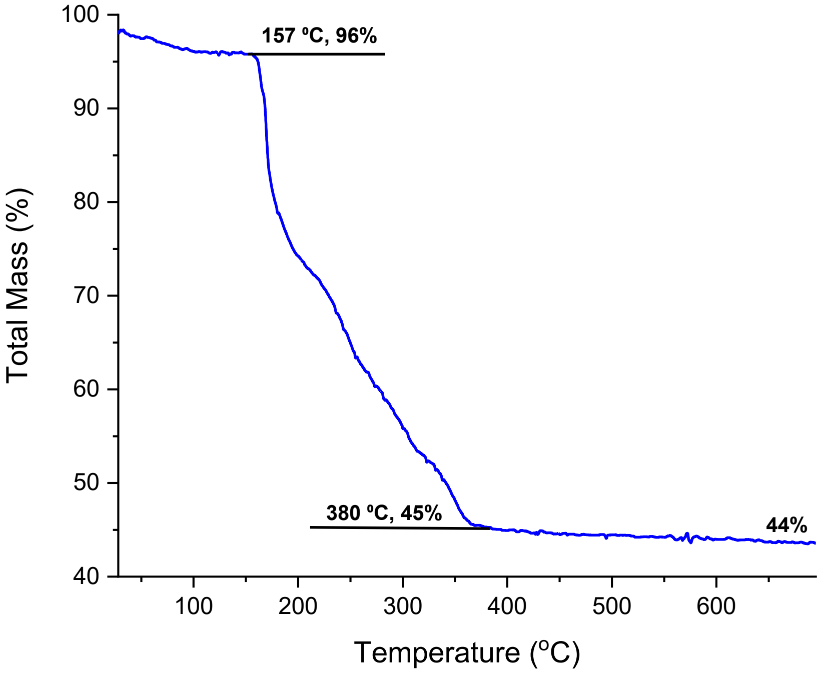

Figure S40. *Thermogravimetric analysis of **1b-AuNPs**. Overall organic ligand content is estimated to be 52%.*

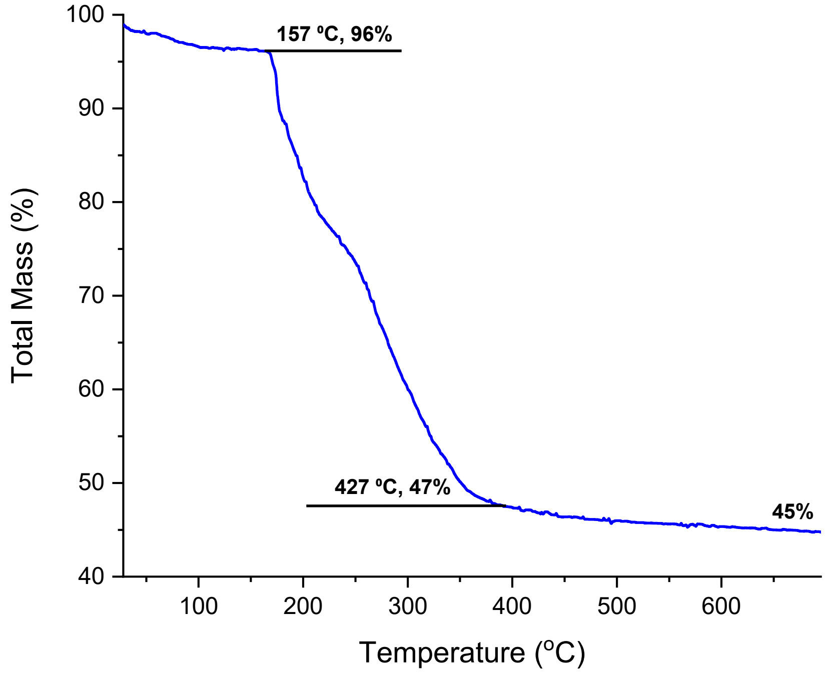

Figure S41. *Thermogravimetric analysis of* ***1c-AuNPs****. Overall organic ligand content is estimated to be 51%.*

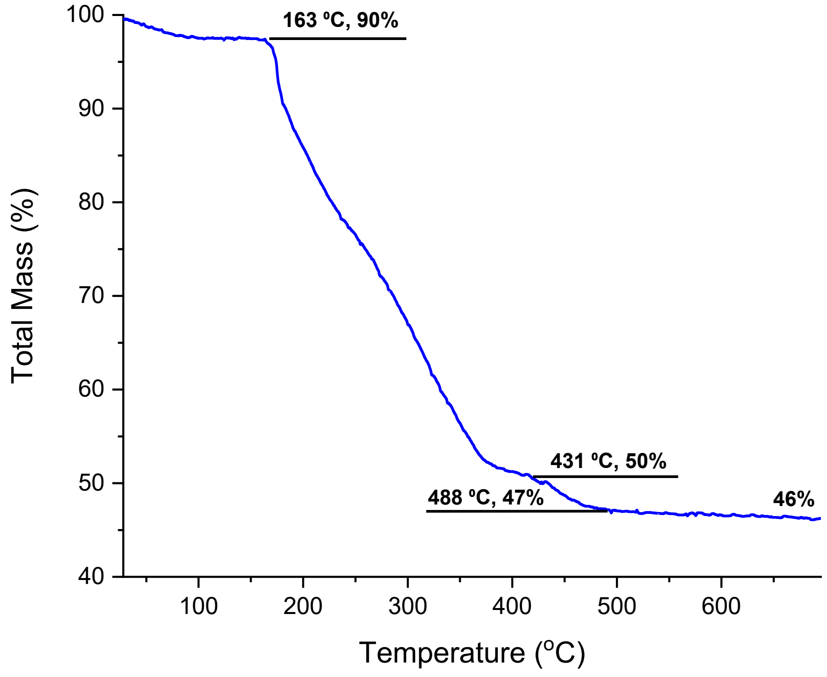

Figure S42. *Thermogravimetric analysis of* ***1d-AuNPs****. Overall organic ligand content is estimated to be 44%.*

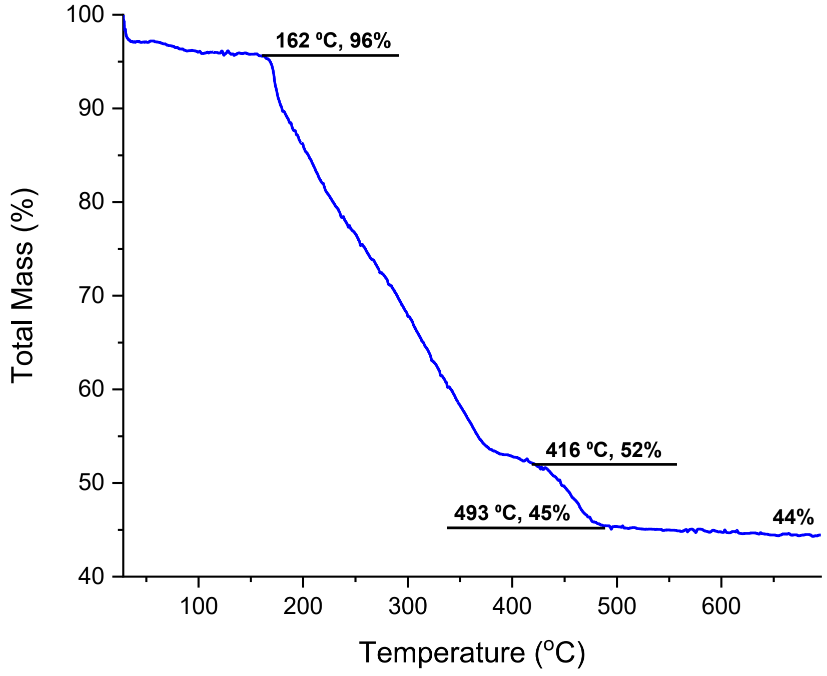

Figure S43. *Thermogravimetric analysis of **1e-AuNPs**. Overall organic ligand content is estimated to be 52%.*

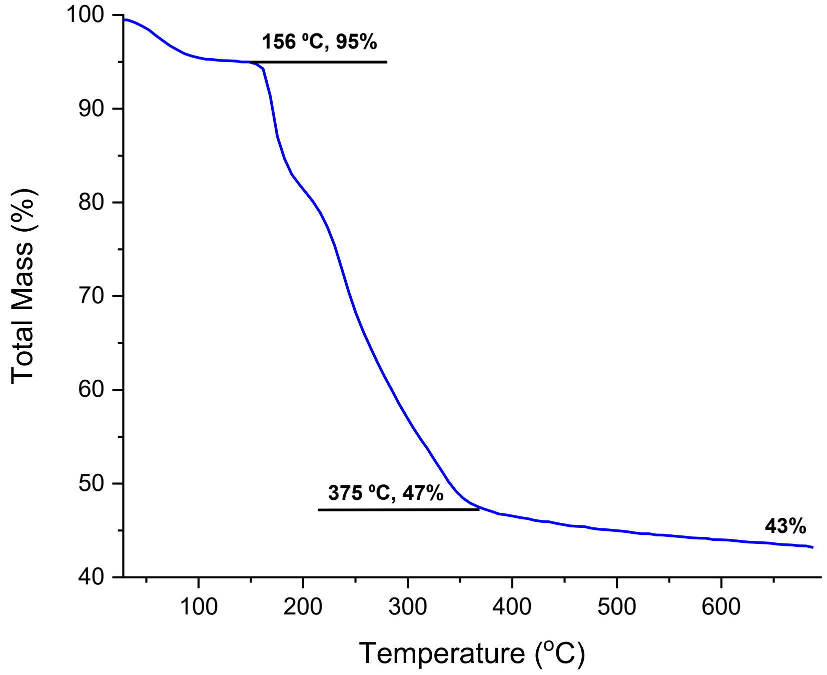

Figure S44. *Thermogravimetric analysis of **2a-AuNPs**. Overall organic ligand content is estimated to be 52%.*

UV-Vis Stability Studies of 2a-AuNPs

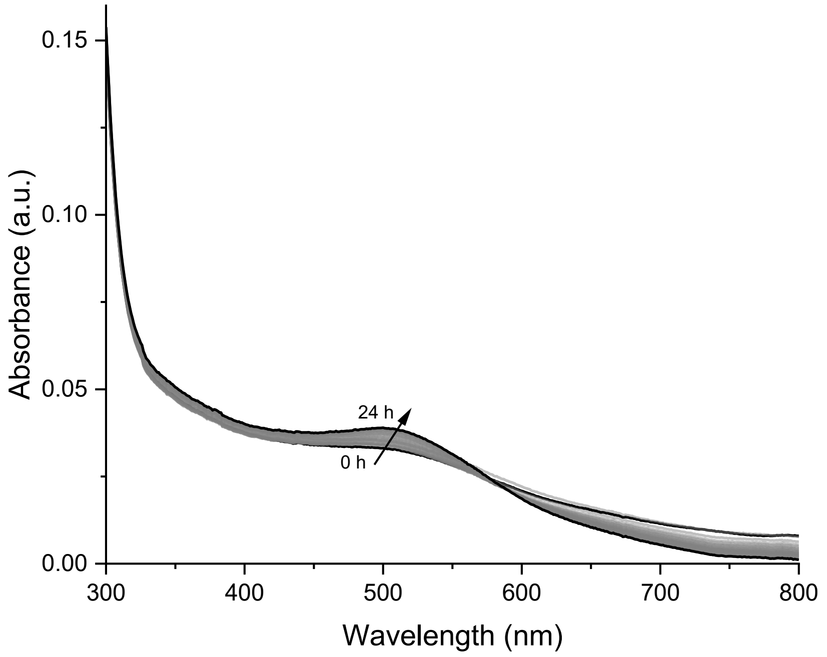

Figure S45. *UV-Vis stability study of* ***2a-AuNPs*** *in MilliQ H_2O over 24 h at room temperature.*

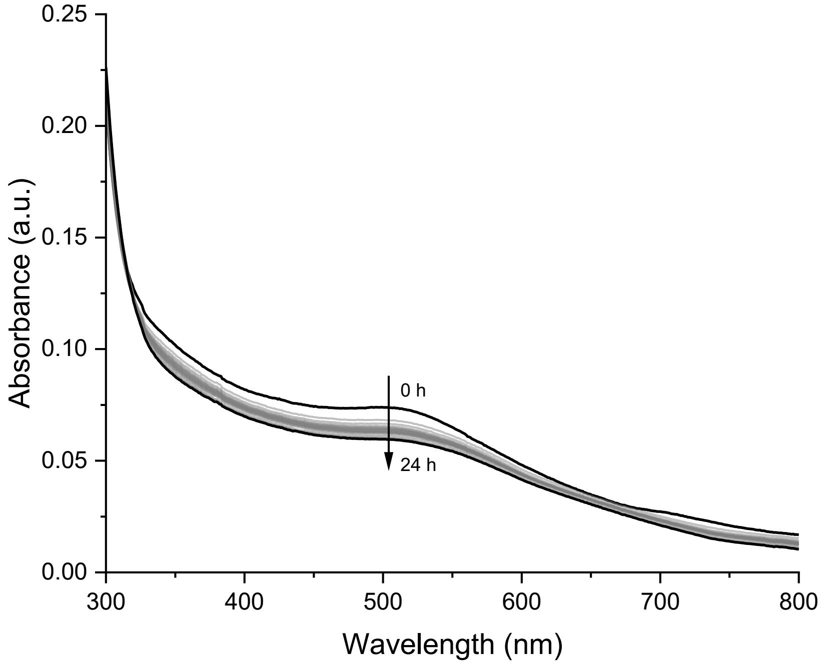

Figure S46. *UV-Vis stability study of* ***2a-AuNPs*** *in aqueous solution of glutathione (GSH, 2mM) over 24 h at room temperature.*

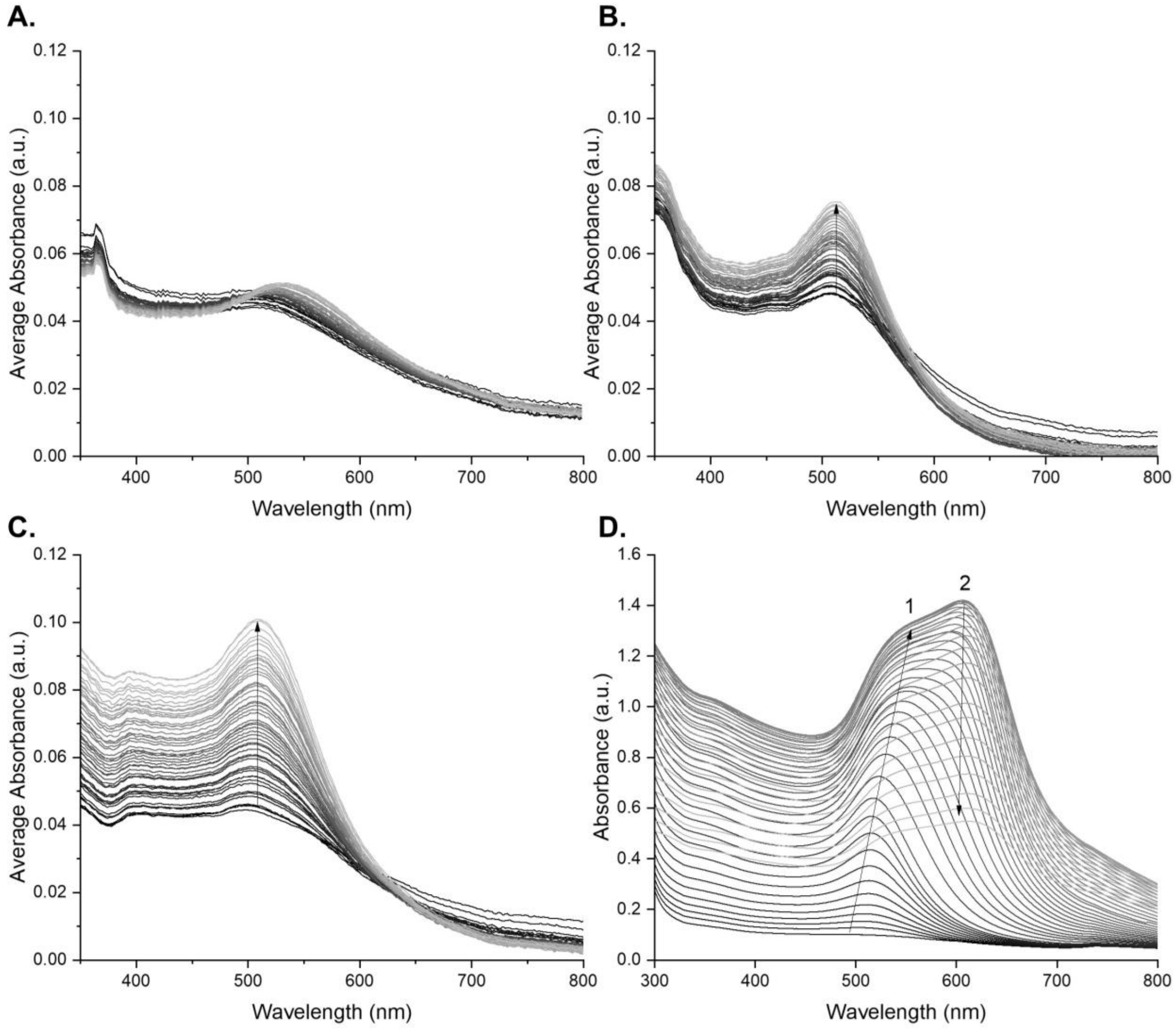

Figure S47. *UV-Vis stability study over 48 h of* ***2a-AuNPs*** *in* ***A.*** *PBS 1x;* ***B.*** *aqueous solution of HCl (pH 3);* ***C.*** *aqueous solution of NaOH (pH 10), all carried out at room temperature; and* ***D.*** *Milli-Q water at 50 °C (arrow 1 from t = 0 to 34 h, arrow 2 from t = 35 h to 48 h).*

Catalytic Reduction of 4-Nitrophenol with ADC-AuNPs

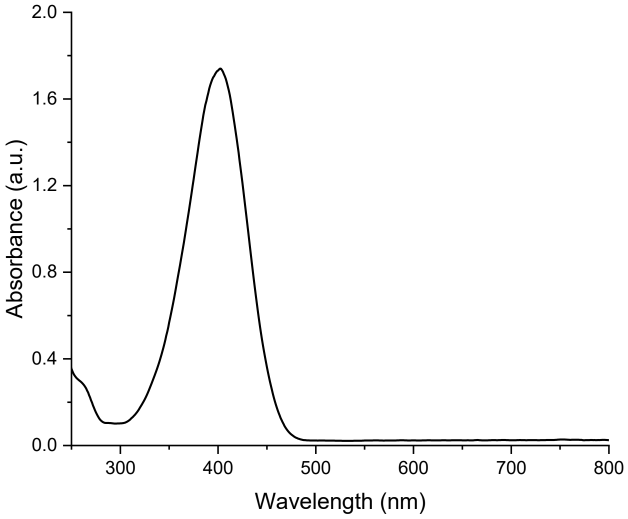

Figure S48. *UV-Vis absorbance spectrum of 4-nitrophenolate after addition of $NaBH_4$ aqueous solution (30 mM) to a solution of 4-nitrophenol in MilliQ H_2O (0.1 mM).*

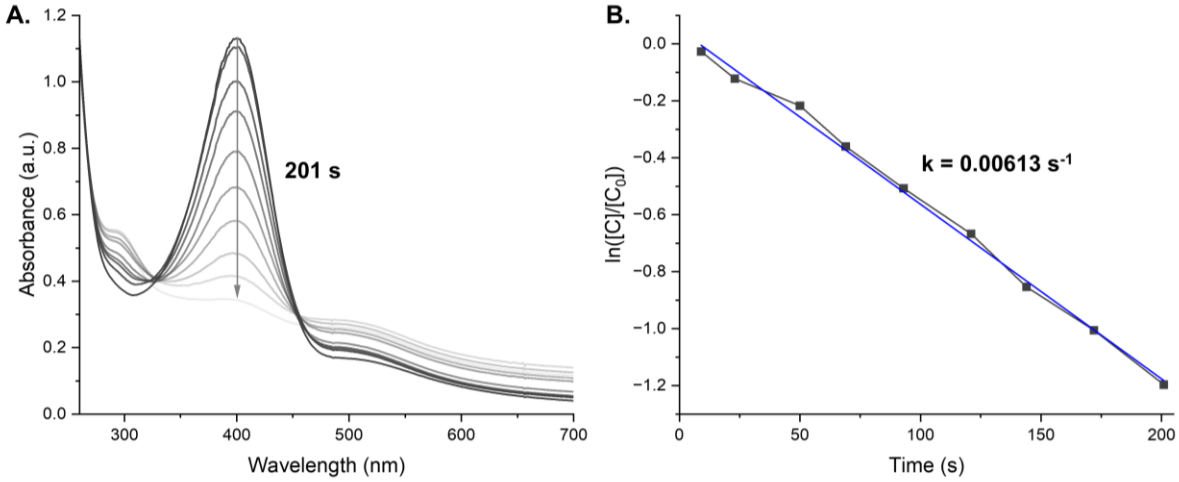

Figure S49. A. *UV-Vis absorption kinetic studies for the reduction of 4-nitrophenol (C_0 = 0.05 mM) catalyzed by **1a-AuNPs** in MeOH/H_2O at r.t.* ***B.*** *Plot of ln([C]/[C_0]) vs. time for **1a-AuNPs** showing first-order kinetics, and calculated rate constant.*

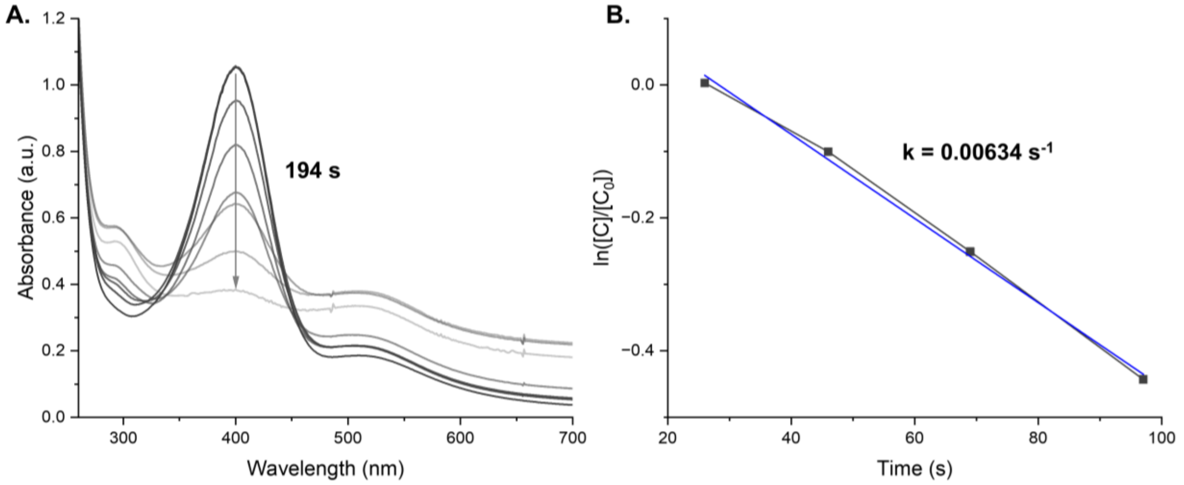

Figure S50. A. *UV-Vis absorption kinetic studies for the reduction of 4-nitrophenol (C_0 = 0.05 mM) catalyzed by* ***1b-AuNPs*** *in MeOH/H_2O at r.t.* ***B.*** *Plot of ln([C]/[C_0]) vs. time for* ***1b-AuNPs*** *showing first-order kinetics, and calculated rate constant.*

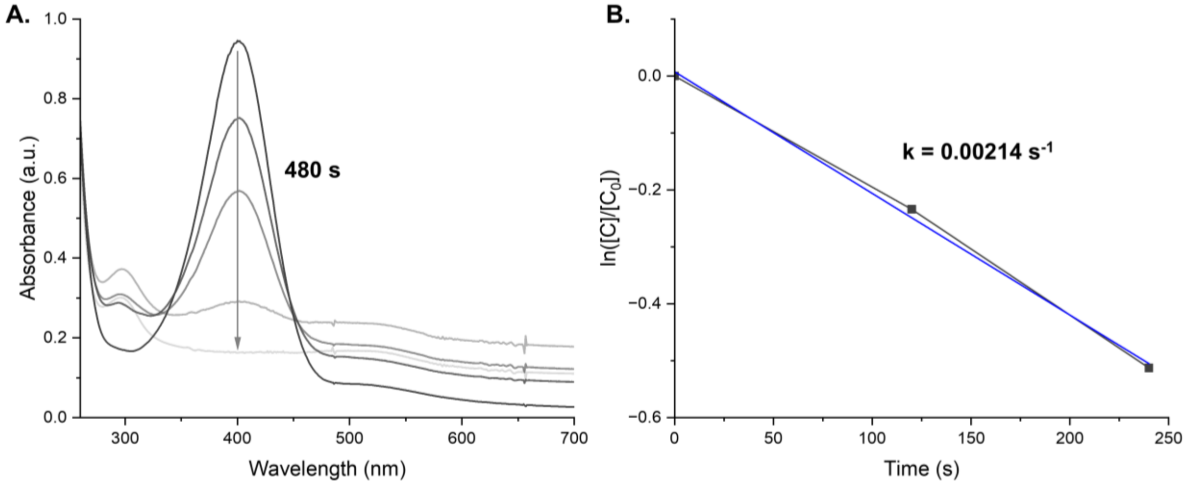

Figure S51. A. *UV-Vis absorption kinetic studies for the reduction of 4-nitrophenol (C_0 = 0.05 mM) catalyzed by* ***1c-AuNPs*** *in MeOH/H_2O at r.t.* ***B.*** *Plot of ln([C]/[C_0]) vs. time for* ***1c-AuNPs*** *showing first-order kinetics, and calculated rate constant.*

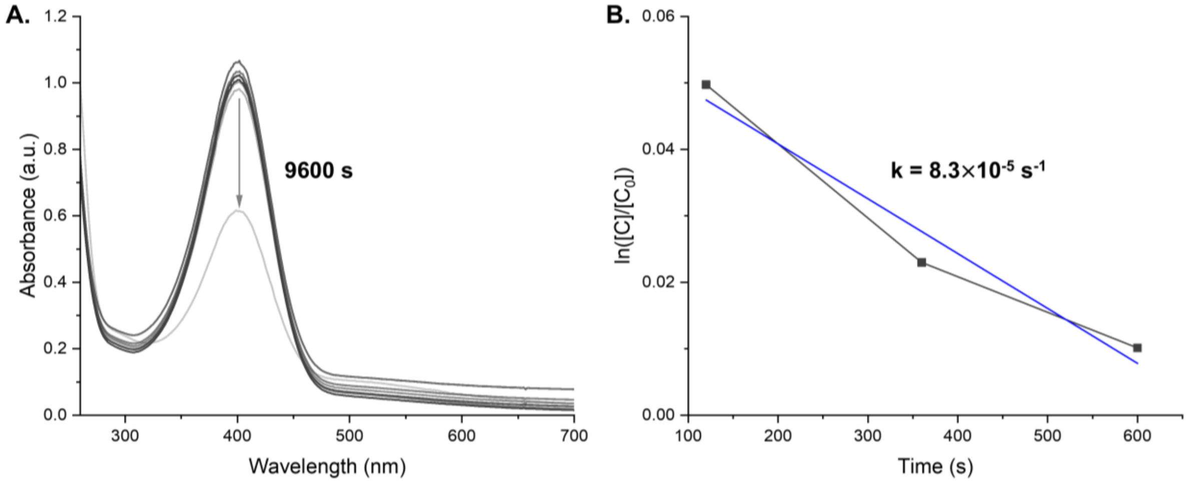

Figure S52. A. *UV-Vis absorption kinetic studies for the reduction of 4-nitrophenol (C_0 = 0.05 mM) catalyzed by* ***1d-AuNPs*** *in MeOH/H_2O at r.t.* ***B.*** *Plot of ln([C]/[C_0]) vs. time for* ***1d-AuNPs*** *showing first-order kinetics, and calculated rate constant.*

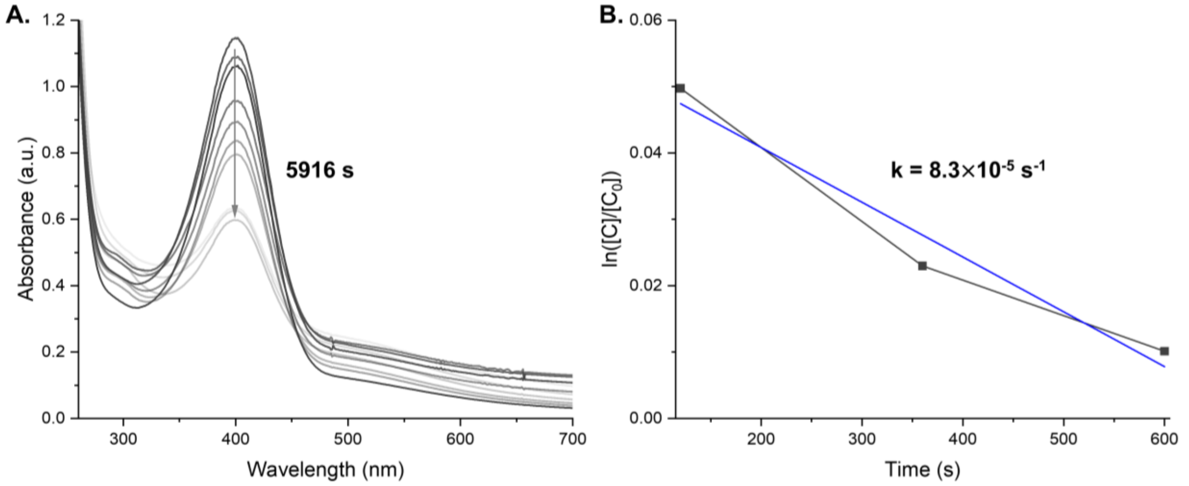

Figure S53. A. *UV-Vis absorption kinetic studies for the reduction of 4-nitrophenol (C_0 = 0.05 mM) catalyzed by* ***1e-AuNPs*** *in MeOH/H_2O at r.t.* ***B.*** *Plot of ln([C]/[C_0]) vs. time for* ***1e-AuNPs*** *showing first-order kinetics, and calculated rate constant.*

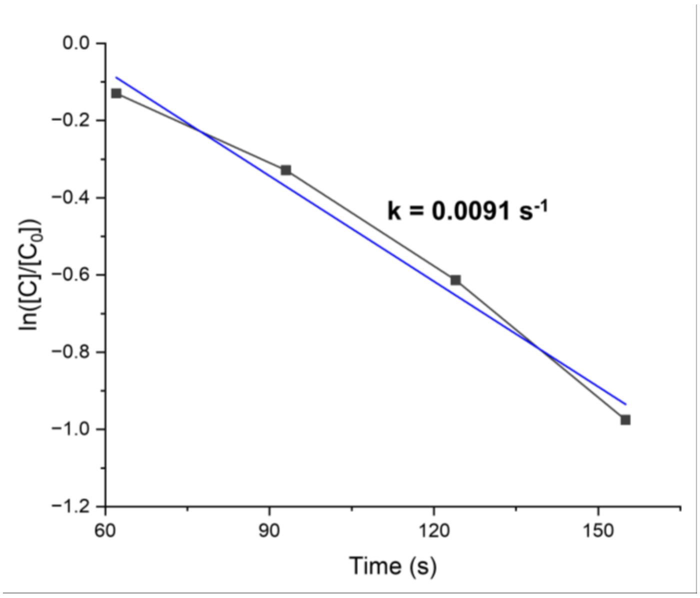

Figure S54. *Plot of ln([C]/[C_0]) vs. time for* ***2a-AuNPs*** *showing first-order kinetics, and calculated rate constant.*

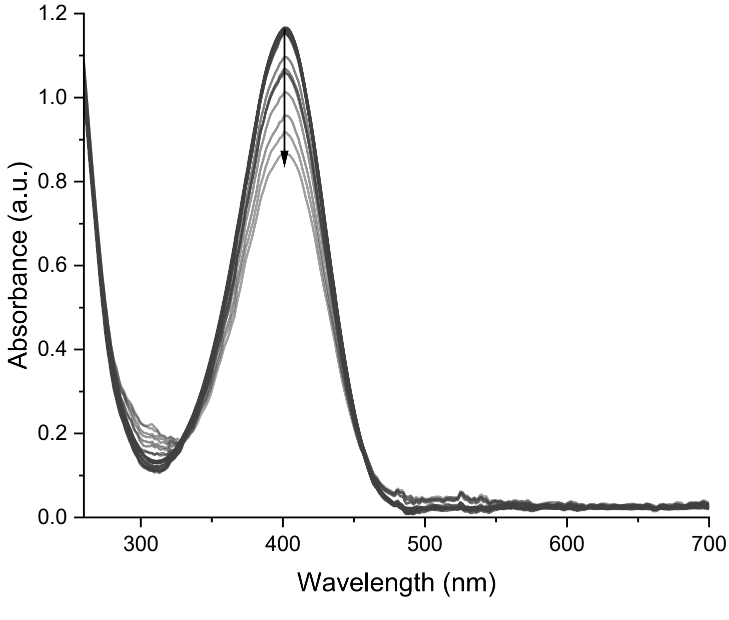

Figure S55. *UV-Vis absorption kinetic studies for the reduction of 4-nitrophenol (C_0 = 0.05 mM) catalyzed by* ***2a*** *in H_2O at r.t. over 10 min.*

Tables

Table S1. *Peak Binding Energies (BE) (eV) and Full Width at Half-Maximum (FWHM) values (eV) for* ***1a*** *and* ***1a-AuNPs*** *obtained by XPS analysis.*

**1a**		
**Name**	**Peak BE (eV)**	**FWHM (eV)**
N 1s	399.99	1.97
C 1s	284.85	1.45
C 1s	286.76	1.17
Au(I) 4f	88.88	1.68
Au(I) 4f	85.20	1.68
**1a-AuNPs**		
**Name**	**Peak BE (eV)**	**FWHM (eV)**
N 1s	399.73	1.44
C 1s	284.84	1.47
C 1s	286.42	1.43
Au(I) 4f	88.87	1.38
Au(I) 4f	85.21	1.31
Au(0) 4f	87.01	1.36
Au(0) 4f	83.35	1.33
